# Polyprenylated acylphloroglucinols from *Garcinia* species and structural revision of seven analogues

**DOI:** 10.1007/s13659-025-00519-6

**Published:** 2025-05-26

**Authors:** Yong-Ge Fu, Yi-Qi Huang, Zhi-Hong Xu, Xia Liu, Xing-Wei Yang

**Affiliations:** 1 School of Pharmaceutical Sciences (Shenzhen), Sun Yat-Sen University, Shenzhen, 518107 People’s Republic of China; 2 Department of Pharmacy, Chongqing Traditional Chinese Medicine Hospital, Chongqing, 400021 China

**Keywords:** *Garcinia xanthochymus*, *Garcinia subelliptica*, Polyprenylated acylphloroglucinol, Structural revision, Cytotoxicity

## Abstract

Our continuous study of the fruits of *Garcinia xanthochymus* and *Garcinia subelliptica* led to the isolation and structural characterization of six new polyprenylated acylphloroglucinols, xanthochymusones N and O (**1** and **2**), (–)-garciyunnanin L (**3**), and garsubelones C–E (**4**–**6**), together with two known analogues. Their structures were elucidated by interpretation of NMR and MS spectroscopic data. It was found that the Grossman-Jacobs rule is no longer applicable to determination of the C-7 configuration of compounds **1**–**3**, as they possess a complex 6/6/6/6/6 fused ring system. The inhibitory activities of all the compounds against two human hepatocellular carcinoma cell lines Huh-7 and HepG2 were evaluated, and compound **1** exhibited moderate cytotoxic activities against HepG2 cells with IC_50_ value 7.3 μM. Furthermore, the previous assignments of some polyprenylated acylphloroglucinols have been proved to be incorrect in this study, and analysis of NMR data enabled the structural revision of seven analogues: hyperselancins A and B, garcinielliptones F and G, garxanthochins A and B, and 13,14-didehydroxygarcicowin C. The revised structures of garcinielliptone F and garxanthochin A were shown to have the same structures of garsubelone B and xanthochymusone K, respectively, and the revised structures of other five compounds have not been reported.

**Graphical abstract:**

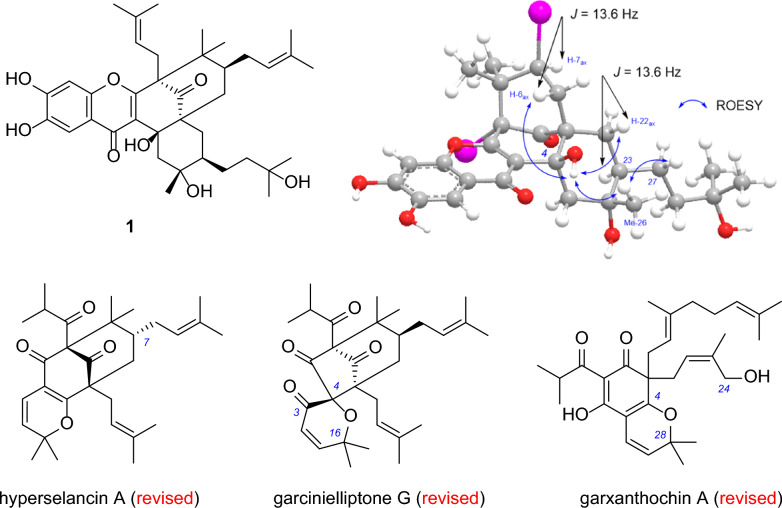

**Supplementary Information:**

The online version contains supplementary material available at 10.1007/s13659-025-00519-6.

## Introduction

**Fig. 1:**
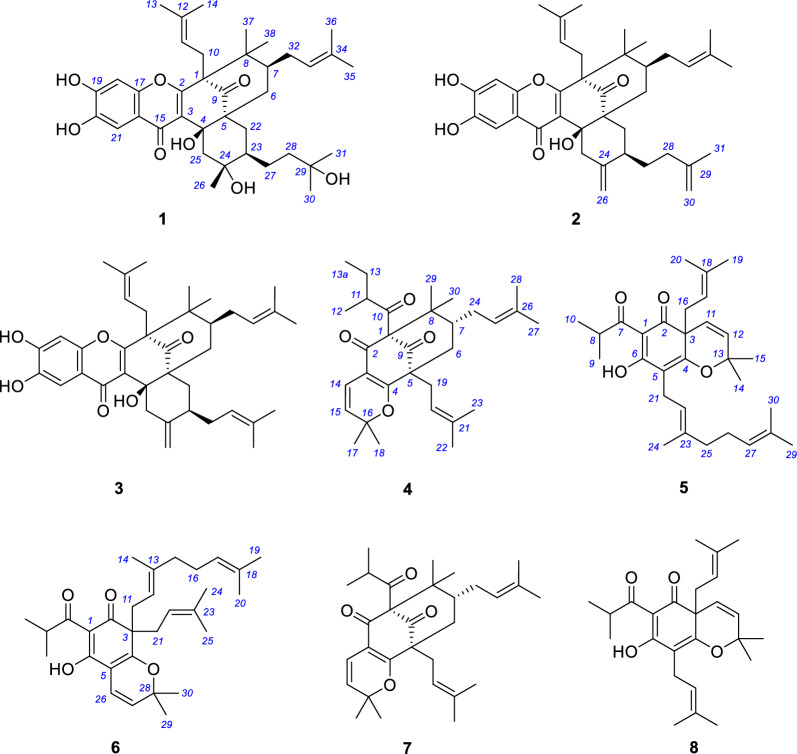
Structures of compounds **1**‒**8**

The genus *Garcinia* comprises more than 300 species and belongs to the family Clusiaceae [1]. This genus is a rich source of secondary metabolites including flavonoids, xanthones, and polyprenylated acylphloroglucinols with diverse bioactivities such as antioxidant, antibacterial, anti-inflammatory, and anticancer activities [1, 2]. We have a long-standing interest in the investigation of structures and bioactivities of polycyclic polyprenylated acylphloroglucinols (PPAPs) [3–14], a special kind of hybrid natural products that are found only in plants of families Clusiaceae and Hypericaceae [3, 15]. Our previous study on *Garcinia xanthochymus* involves the structural characterizations of a series of type B PPAPs sharing a lavandulyl-derived substituent as well as some simple polyprenylated acylphloroglucinols derivatives [14, 16]. In a continuous search for new and bioactive natural polyprenylated acylphloroglucinols, the phytochemical investigation of the fruits of *G. xanthochymus* and *G. subelliptica* were further carried out. As a result, three new type B PPAPs, xanthochymusones N and O (**1** and **2**), (–)-garciyunnanin L (**3**), were isolated from *G. xanthochymus* (Fig. 1). A new type A PPAP, garsubelone C (**4**), and two new simple polyprenylated acylphloroglucinols, garsubelones D and E (**5** and **6**), together with two known analogues (**7** and **8**), were isolated from *G. subelliptica* (Fig. 1). Herein are described the isolation and structural elucidation of the new prenylated acylphloroglucinols and the inhibitory activity of all compounds obtained against two human hepatocellular carcinoma cells Huh-7 and HepG2. Also, the previous assignments of some polyprenylated acylphloroglucinols have been proved to be incorrect. Detailed analysis of NMR and other spectroscopic data enabled the structural revision of hyperselancins A and B, garcinielliptones F and G, garxanthochins A and B, and 13,14-didehydroxygarcicowin C.

## Results and discussion

### Structural characterization of new polyprenylated acylphloroglucinols

The MeOH extracts of the fruits of *G. xanthochymus* and *G. subelliptica* were subjected to several purification steps. Six new polyprenylated acylphloroglucinols, xanthochymusones N and O (**1** and **2**), (–)-garciyunnanin L (**3**), garsubelones C–E (**4**–**6**), and two known analogues were obtained (Fig. 1). Known compounds were identified as garsubelone B (**7**) [17] and lupulone B (**8**) [18] by comparison of the physical and chemical data to the reported literature. Compound **3** were proven to be enantiomer of ( +)-garciyunnanin L [19] via NMR, optical rotation, and ECD data.

**Fig. 2:**
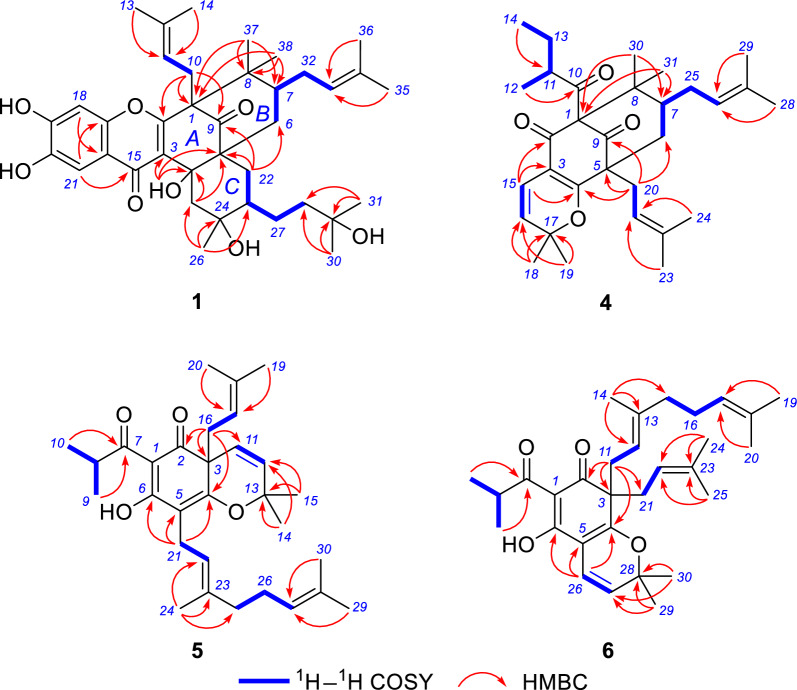
Key ^1^H–^1^H COSY and HMBC correlations of compounds **1** and **4**–**6**

**Fig. 3:**
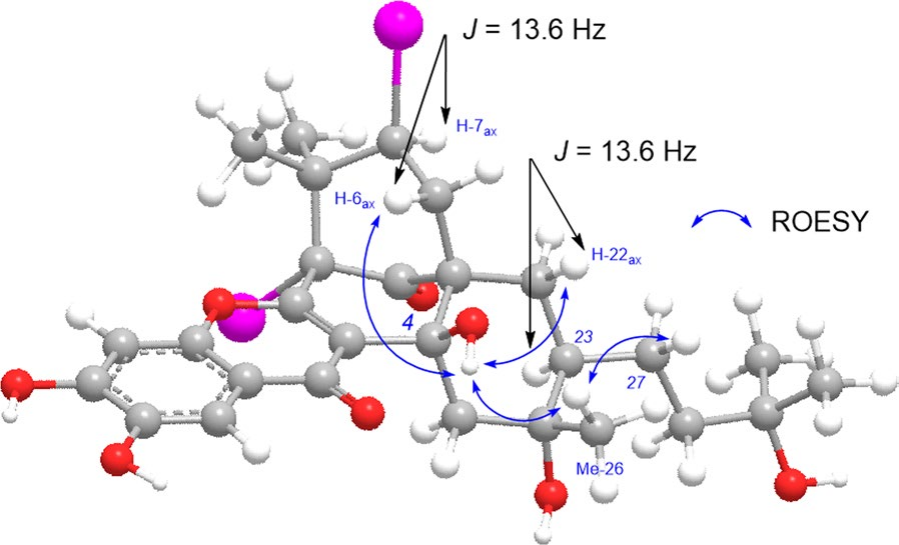
Simplified and configuration optimized molecular model of **1**. Black arrows, coupling constants; blue arrows, NOE correlations

Xanthochymusone N (**1**) was isolated as a yellow gum. Its molecular formula, C_38_H_52_O_8_, was established by analysis of ^13^C NMR (Table 1) and HRESIMS data (*m/z* 635.3586, [M – H]^−^, cacld for 635.3584). The UV spectrum revealed the presence of conjugated groups with maximum absorptions at 232, 293, and 327 nm. The FTIR spectrum displayed absorption bands due to hydroxy (3400 cm^−1^), carbonyl (1724 cm^−1^), and olefinic (1621 and 1467 cm^−1^) functionalities. The ^1^H NMR spectrum (Table 1) exhibited signals of two singlet aromatic protons (*δ*_H_ 7.40 and 6.78, s), olefinic protons (*δ*_H_ 4.92 and 4.00), and nine singlet methyl groups (*δ*_H_ 0.52 ~ 1.65). The ^13^C and DEPT-NMR data revealed a total of 38 carbon resonances (Table 1), including a nonconjugated carbonyl (*δ*_C_ 209.2), a group of enol carbons at *δ*_C_ 122.4 and 164.7, six quaternary carbons (*δ*_C_ 75.8, 73.2, 72.8, 60.9, 54.1, and 49.5), two methines (*δ*_C_ 43.4 and 41.6). The above signals, in combination with a number of PPAPs that have been isolated from this plant, [13, 20–26] indicated that compound **1** was a PPAP derivative. Comparison of its 1D NMR data with those of garcinialone [27], a type B PPAP with 6/6/6/6/6 fused ring system, indicated that they were structurally similar. The core carbon system of **1** was confirmed by the correlations from *δ*_H_ 0.99 and 0.92 (Me-37 and Me-38) to *δ*_C_ 60.9 (C-1), 43.4 (C-7), and 49.5 (C-8), from *δ*_H_ 2.82 and 2.37 (H_2_-10) to C-1 and *δ*_C_ 164.7 (C-2) and 209.2 (C-9), from a hydroxyl proton at *δ*_H_ 5.44 (4-OH) to *δ*_C_ 122.4 (C-3), 75.8 (C-4), and 54.1 (C-5), from *δ*_H_ 1.97 and 1.36 (H_2_-22) to C-5, C-9, and *δ*_C_ 38.5 (C-6), and from *δ*_H_ 1.59 (Me-26) to *δ*_C_ 41.6 (C-23), 73.8 (C-24), and 51.6 (C-25) in the HMBC spectrum, in combination with the correlations of *δ*_H_ 1.04 (H-7)/2.32 and 1.44 (H_2_-6), and *δ*_H_ 2.33 (H-23)/H_2_-22 in the ^1^H–^1^H COSY spectrum (Fig. 2). An oxidized isoprenyl group linked to C-23 was further determined via HBMC correlations of *δ*_H_ 1.37 and 1.39 (Me-30 and Me-31) with *δ*_C_ 72.8 (C-29) and 40.1 (C-28), coupled with the proton spin system of H-23/*δ*_H_ 1.83 and 1.34 (H_2_-27)/*δ*_H_ 2.07 and 1.73 (H_2_-28) in the ^1^H–^1^H COSY spectrum. Other parts of the planer structure of **1** were confirmed to be identical to those of garcinialone by detailed analysis of the 2D NMR spectroscopic data (Fig. 2). The relative configuration of **1** could be defined by using a combination of ^1^H–^1^H coupling constants, conformational analysis, NOE experiments, as well as biosynthetic consideration. As described in the literature, these rare PPAPs could be biosynthetically generated from type B PPAPs with a bicyclo[3.3.1]nonane-2,4,9-trione core carrying a lavandulyl substituent by Alder-ene reaction [19, 27]. As the cyclization took place from the less hindered *α*-phase, the resulting *β*-axial hydroxyl group (4-OH) was formed. In the ^1^H spectrum of **1** (measured in CDCl_3_), the ^3^* J* coupling constants of both H-6a (*δ*_H_ 2.32, t, *J* = 13.6 Hz) and H-22b (*δ*_H_ 1.36, t, *J* = 13.6 Hz) were 13.6 Hz. So, H-6a, H-7, H-22b, and H-23 were axial while the C-7 prenyl group and C-23 substituent were equatorial. Then, the NOE contacts of 4-OH with H-6_ax_, H-22_ax_, and Me-26 indicated that the relative configuration of C-7 and C-23, and C-24 (Fig. 3). The NOE correlation of Me-26 (*δ*_H_ 1.33) with H-27a (*δ*_H_ 1.85) measured in CD_3_OD also confirmed the relative configuration of C-23 and C-24. The above deduction suggested that B ring adopted boat conformation while C ring adopted chair conformations.

**Table 1:** ^1^H (600 MHz) and ^13^C (150 MHz) NMR data of compounds **1** and **2**

No.	**1**^a^		**1**^b^		**2**^a^	
	*δ*_C_, type	*δ*_H_ mult. (*J* in Hz)	*δ*_C_, type	*δ*_H_ mult. (*J* in Hz)	*δ*_C_, type	*δ*_H_ mult. (*J* in Hz)
1	60.9, C		62.2, C		61.5, C	
2	164.7, C		165.1, C		165.6, C	
3	122.4, C		123.4, C		122.7, C	
4	75.8, C		77.7, C		77.4, C	
4-OH		5.44, s				
5	54.1, C		55.4, C		54.9, C	
6	38.5, CH_2_	2.32, t (13.6)	39.9, CH_2_	2.39, t (13.8)	39.3, CH_2_	2.41, t (14.0)
		1.44, dd (13.6, 2.9)		1.43, dd (13.8, 3.1)		1.48, m
7	43.4, CH	1.04, m	45.1, CH	1.06, m	43.8, CH	1.11, m
8	49.5, C		50.3, C		49.6, C	
9	209.2, C		211.3, C		210.3, C	
10	27.0, CH_2_	2.82, dd (13.4, 7.5)	28.2, CH_2_	2.77, brd (13.6)	27.4, CH_2_	2.85, dd (13.5, 4.2)
		2.37, dd (13.4, 7.5)		2.71, dd (13.6, 9.6)		2.61, m
11	118.5, CH	4.00, t (7.5)	120.4, CH	4.54, m	118.8, CH	4.47, t (6.3)
12 16	135.2, C		135.4, C		135.0, C	
13	24.2, CH_3_	0.52, s	25.9, CH_3_	1.36, s	26.0, CH_3_	1.37, s
14	17.9, CH_3_	1.46, s	18.4, CH_3_	1.62, s	18.3, CH_3_	1.60, s
15	178.4, C		179.6, C		178.6, C	
16	115.6, C		116.8, C		116.4, C	
17	152.8, C		155.0, C		152.4, C	
18	101.8 CH	6.78, s	103.4, CH	6.86, s	102.8, CH	6.94, s
19	151.3, C		152.5, C		151.7, C	
20	143.5, C		146.5, C		143.3, C	
21	107.9, CH	7.40, s	108.1, CH	7.36, s	108.2, CH	7.72, s
22	34.4, CH_2_	1.97, dd (13.6, 3.1)	34.1, CH_2_	2.09, dd (13.6, 3.5)	37.0, CH_2_	2.25, m
		1.36, t (13.6)		1.19, t (13.6)		1.26, overlap
23	41.6, CH	2.33, overlap	44.9, CH	2.02, m	38.3, CH	2.61, overlap
24	73.8, C		73.7, C		147.1, C	
25	51.6, CH_2_	2.43, d (13.8)	52.2, CH_2_	2.47, d (14.0)	47.4, CH_2_	2.97, d (13.7)
		0.92, d (13.8)		1.20, d (14.0)		1.83, d (13.7)
26	24.0, CH_3_	1.59, s	23.4, CH_3_	1.33, s	109.2, CH_2_	4.88, s
						4.84, s
27	26.0, CH_2_	1.83, m	25.3, CH_2_	1.85, m	30.6, CH_3_	1.87, m
		1.34, m		0.99, m		1.46, overlap
28	40.1, CH_2_	2.07, m	43.1, CH_2_	1.76, m	35.6, CH_2_	2.21, m
		1.73, m		1.53, m		2.17, m
29	72.8, C		71.8, C		146.7, C	
30	30.6, CH_3_	1.37, s	29.5, CH_3_	1.23, s	109.6, CH_2_	4.75, s
						4.74, s
31	29.2, CH_3_	1.39, s	28.9, CH_3_	1.22, s	22.9, CH_3_	1.78, s
32	28.6, CH_2_	1.90, brd (13.4)	29.9, CH_2_	1.96, brd (14.0)	28.8, CH_2_	1.94, brd (13.4)
		1.55, m		1.64, m		1.57, m
33	122.9, CH	4.92, t (6.4)	124.4, CH	4.96, t (6.8)	122.9, CH	4.93, t (6.5)
34	133.1, C		134.1, C		133.5, C	
35	25.8, CH_3_	1.65, s	26.0, CH_3_	1.67, s	26.0, CH_3_	1.66, s
36	17.9, CH_3_	1.49, s	18.0, CH_3_	1.52, s	18.0, CH_3_	1.49, s
37	25.0, CH_3_	0.99, s	25.5, CH_3_	1.03, s	25.2, CH_3_	1.04, s
38	20.1, CH_3_	0.92, s	20.5, CH_3_	1.03, s	20.2, CH_3_	1.00, s

^a^Recorded in CDCl_3_

^b^Recorded in methanol-*d*_4_

It is worth noting that the chemical shift of C-7 (*δ*_C_ 43.4) and the difference in chemical shifts of the two H-6 protons (0.88 ppm) and the configurational assignment of C-7 substituent (endo) is against the Grossman-Jacobs rule of determination of the C-7 configuration [3, 15]. The fact may be ascribable to the conformational change of B ring (from chair to boat) after the Alder-ene reaction (Fig. 3). So, the Grossman-Jacobs rule is no longer applicable to determination of the C-7 configuration of this kind of PPAPs.

Xanthochymusone O (**2**) was assigned the molecular formula C_38_H_48_O_6_ by analysis of its ^13^C NMR and HREIMS data. The ^1^H and ^13^C NMR data of **2** (Table 1) resembled those of garciyunnanin L [19]. The structure of **2** was shown to be a double-bond regioisomer of garciyunnanin L (*Δ*^29,30^ in **2** and *Δ*^28,29^ in garciyunnanin L). The 2D NMR data showed that the other structural features of **2** were identical to those of garciyunnanin L (Fig. 2).

The ^1^H and ^13^C NMR spectra of **3** are identical to those of ( +)-garciyunnanin L [19]. Nevertheless, the opposite optical rotations of **3** and ( +)-garciyunnanin L ([*α*]_D_ = –21 and + 28.5, respectively) and opposite experimental CD curves indicate that these compounds are enantiomeric. Considering that compounds **1**–**3** are co-isolates of *G. xanthochymus* and their absolute configurations should be consistent with those of xanthochymusones reported previously [14].

Garsubelone C (**4**) has NMR spectra nearly identical to garsubelone B (**7**) [17], except the isopropyl group in garsubelone B was replaced by a sec-butyl group (*δ*_C_ 49.5, C-11; 17.8, C-12; 26.7, C-13; and 11.7, C-13a) (Table 2), so we assign to garsubelone C the structure **4**. The HMBC correlation of Me-12 (*δ*_H_ 1.02, d, *J* = 6.5 Hz) with C-10 (*δ*_C_ 208.6) and the correlations of Me-12/*δ*_H_ 1.78 (H-11)/1.95 and 1.32 (H_2_-13)/*δ*_H_ 0.81 (Me-13a) in the ^1^H–^1^H COSY spectrum confirmed the assignment (Fig. 2). Since the absolute configuration of garsubelone B was undoubtfully determined as 1*R*,5*S*,7*R* on the basis of X-ray crystallographic data and garsubelones B and C are isolated from the same plant (*G. subelliptica*) [17], the absolute configurations of C-1, C-5, and C-7 for compound **4** should be the same as those of garsubelone B.

**Table 2:** ^1^H and ^13^C NMR data for **4** and hyperselancins A and B in CDCl_3_

No.	**4**		**Hyperselancin A**^a^		**Hyperselancin B**^a^	
	*δ*_C_, type	*δ*_H_ mult. (*J* in Hz)	*δ*_C_, type	*δ*_H_ mult. (*J* in Hz)	*δ*_C_, type	*δ*_H_ mult. (*J* in Hz)
1	83.6, C		81.2, C		81.1, C	
2	188.3, C		188.8, C		187.6, C	
3	114.5, C		114.1, C		114.2, C	
4	170.7, C		171.7, C		171.8, C	
5	56.8, C		55.1, C		55.0, C	
6	39.4, CH_2_	1.90, dd (13.2, 3.8)	38.4, CH_2_	**2.11**, m	38.1, CH_2_	**2.11**, m
		1.39, t (13.2)		**2.06**, m		**2.06**, m
7	43.5, CH	1.47, m	**47.7**, CH	1.36, m	**47.7**, CH	1.36, m
8	47.0, C		48.2, C		48.0, C	
9	206.4, C		206.8, C		206.7, C	
10	208.6, C		208.6, C		208.9, C	
11	49.5, CH	1.78, m	48.9, CH	1.84, m	41.9, CH	2.15, m
12 16	17.8, CH_3_	1.02, d (6.5)	18.0, CH_3_	1.02, d (6.1)	21.7, CH_3_	1.03, d (6.6)
13	26.7, CH_2_	1.95, m	26.7, CH_2_	1.94, m	20.6, CH_3_	1.14, d (6.6)
		1.32, m		1.32, m		
13a	11.7, CH_3_	0.81, t (7.5)	11.7, CH_3_	0.83, t (7.5)		
14	115.5, CH	6.48, d (10.0)	115.5, CH	6.49, d (10.1)	115.4, CH	6.49, d (10.1)
15	123.7, CH	5.35, d (10.0)	124.0, CH	5.38, d (10.1)	124.0, CH	5.38, d (10.1)
16	81.9, C		82.5, C		82.5, C	
17	28.6, CH_3_	1.39, s	29.3, CH_3_	1.51, s	29.3, CH_3_	1.51, s
18	28.4, CH_3_	1.46, s	28.2, CH_3_	1.37, s	28.1, CH_3_	1.36, s
19	29.0, CH_2_	2.50, dd (14.6, 6.1)	30.5, CH_2_	2.45, d (6.6)	30.5, CH_2_	2.45, d (6.6)
		2.41, dd (14.6, 7.6)				
20	119.3, CH	4.98, m	119.7, CH	4.99, t (6.6)	119.7, CH	5.02, t (6.6)
21	133.9, C		133.6, C		133.5, C	
22	26.0, CH_3_	1.64, s	26.0, CH_3_	1.65, s	25.9, CH_3_	1.67, s
23	18.2, CH_3_	1.68, s	18.1, CH_3_	1.69, s	18.1, CH_3_	1.53, s
24	26.6, CH_2_	2.10, m	29.5, CH_2_	2.10, m	29.4, CH_2_	2.10, m
		1.64, m		1.87, m		1.87, m
25	122.7, CH	4.96, m	125.0, CH	4.92, t (7.0)	125.0, CH	4.92, t (7.5)
26	133.3, C		132.1, C		132.1, C	
27	25.7, CH_3_	1.64, s	25.8, CH_3_	1.65, s	25.8, CH_3_	1.65, s
28	17.8, CH_3_	1.54, s	18.1, CH_3_	1.53, s	18.1, CH_3_	1.68, s
29	23.0, CH_3_	1.23, s	26.5, CH_3_	1.19, s	26.5, CH_3_	1.20, s
30	15.6, CH_3_	0.99, s	22.1, CH_3_	1.32, s	22.1, CH_3_	1.32, s

^a^Data from the literature

The molecular formula of garsubelone D (**5**) was determined as C_30_H_42_O_4_ by analysis of its ^13^C NMR (Table 3) and HRESIMS data (*m/z* 489.2978, [M + Na]^+^), 68 mass units more than that of lupulone B (**8**) [18]. On the basis of analysis of its 1D (Table 3) and 2D NMR data, compound **5** was assigned to possess the same backbone as lupulone B. The structural novelty of **5** involved the presence of a geranyl side chain rather than a prenyl group. The geranyl group linked to C-5 was confirmed by the ^1^H–^1^H COSY correlations of H_2_-21/H-22 and H_2_-25/H_2_-26/H-27, in combination with the HMBC correlations of Me-24 (*δ*_H_ 1.75) with C-22 (*δ*_C_ 123.0) and C-25 (*δ*_C_ 40.9) and of both Me-29 (*δ*_H_ 1.66) and Me-30 (*δ*_H_ 1.56) with C-27 (*δ*_C_ 125.3) (Fig. 2). The NOE contact of Me-24/H_2_-21 in the NOESY spectrum defined the *E* configuration of the corresponding ene.

**Table 3:** ^1^H and ^13^C NMR data for compounds** 5** and **6** in methanol-*d*_4_

No.	**5**		**6**	
	*δ*_C_, type	*δ*_H_ mult. (*J* in Hz)	*δ*_C_, type	*δ*_H_ mult. (*J* in Hz)
1	108.3, C		108.1, C	
2	195.0, C		197.1, C	
3	54.2, C		58.5, C	
4	169.4, C		173.5, C	
5	116.5, C		107.5, C	
6	191.4, C		187.5, C	
7	208.4, C		208.6, C	
8	36.7, CH	3.88, sept (6.8)	36.9, CH	3.96, sept (6.8)
9	19.8, CH_3_	1.14, d (6.8)	19.3, CH_3_	1.10, d (6.8)
10	18.9, CH_3_	1.09, d (6.8)	19.2, CH_3_	1.09, d (6.8)
11	124.1, CH	6.21, d (9.9)	38.7, CH_2_	2.67, m
				2.54, m
12 16	133.3, CH	5.90, d (9.9)	119.0, CH	4.81, m
13	84.0, C		139.7, C	
14	29.0, CH_3_	1.56, s	16.9, CH_3_	1.57, s
15	29.9, CH_3_	1.34, s	40.7, CH_2_	1.84, t (7.2)
16	45.7, CH_2_	2.57, m	27.6, CH_2_	1.91, m
		2.41, m		
17	118.6, CH	4.90, m	125.0, CH	4.95, t (6.9)
18	137.8, C		132.4, C	
19	26.2, CH_3_	1.59, s	26.0, CH_3_	1.59, s
20	18.0, CH_3_	1.48, s	17.7, CH_3_	1.51, s
21	22.0, CH_2_	3.18, dd (14.1, 7.4)	38.7, CH_2_	2.68, m
		3.06, dd (14.1, 7.4)		2.51, m
22	123.0, CH	5.09, m	119.3, CH	4.79, m
23	136.2, C		136.0, C	
24	16.4, CH_3_	1.75, s	25.9, CH_3_	1.58, s
25	40.9, CH_2_	1.97, t (7.7)	18.3, CH_3_	1.56, s
		1.83, m		
26	27.6, CH_2_	2.06, m	115.0, CH	6.42, d (10.1)
		1.89, m		
27	125.3, CH	5.05, m	125.1, CH	5.49, d (10.1)
28	132.6, C		82.9, C	
29	25.9, CH_3_	1.66, s	29.2, CH_3_	1.45, s
30	17.8, CH_3_	1.56, s	29.1, CH_3_	1.43, s

Garsubelone E (**6**) was assigned the molecular formula C_30_H_42_O_4_ by analysis of its ^13^C NMR and HREIMS data. The ^1^H and ^13^C NMR data of **6** (Table 3) resembled those of xanthochymusone K [16]. Instead of a hydroxymethyl in xanthochymusone K, a methyl carbon at *δ*_C_ 18.3 (C-25) appeared in **6**, suggesting that **6** possessed a complete prenyl group. This suggestion was further supported by the correlations of Me-24 (*δ*_H_ 1.58) and Me-25 (*δ*_H_ 1.56) with *δ*_C_ 119.3 (C-22) and 136.0 (C-23), as well as ^1^H–^1^H COSY correlations of *δ*_H_ 2.68 and 2.51 (H_2_-21)/*δ*_H_ 4.79 (H-22). The 2D NMR data showed that the other structural features **6** were identical to those of xanthochymusone K (Fig. 2).

### Structural revision of seven PAPs

**Scheme 1:**
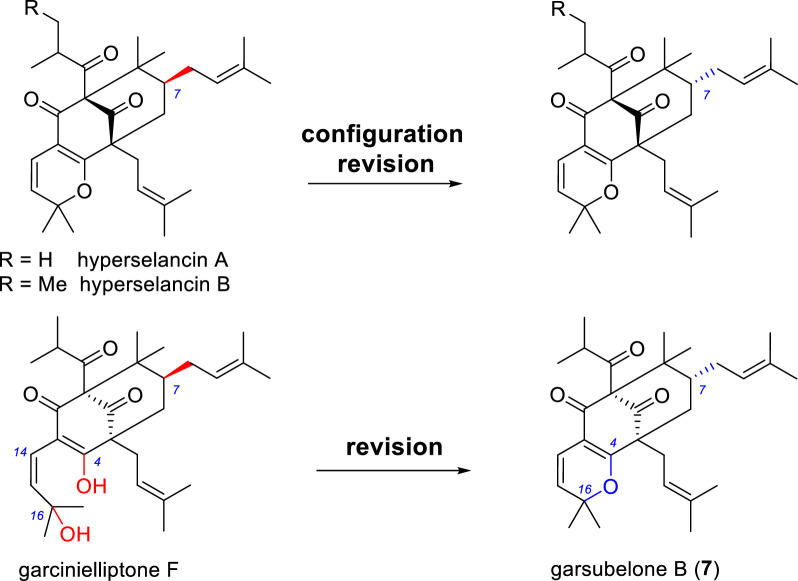
Structural revision of hyperselancins A and B and garcinielliptone F

The previous assignments of some PAPs were controversial and have been proved to be incorrect in this study. Garsubelones B and C have same planar structures and relative configuration to those of hyperselancins A and B (Scheme 1) [28], respectively, however, their reported ^1^H and ^13^C NMR spectroscopic data are different (Table 2). The chemical shift of C-7 (*δ*_C_ 47.7) and the difference in chemical shifts of the two H-6 protons (0.05 ppm) of both hyperselancins A and B indicates that the C-7 substituents of the two compounds should be endo according to the Grossman-Jacobs rule [3, 15]. The original authors also referred to the Grossman-Jacobs rule, but unfortunately they seem to have confused the concept of endo/exo and *α*/*β* [28]. Hence, we revise the relative configuration of C-7 of hyperselancins A and B (Scheme 1).

**Scheme 2:**
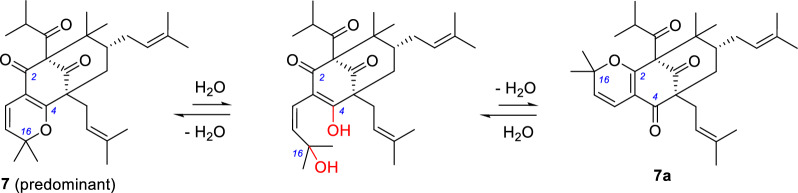
Transformation between **7** and **7a**

Garsubelone B were found to share identical ^1^H and ^13^C NMR data to garcinielliptone F (Table 4) [29, 30], a PPAP resulting from hydrolysis of the enol ether of garsubelone B, suggesting structural reassignment of garcinielliptone F (Scheme 1). The original authors may have misinterpreted the mass spectrometry data. Interestingly, we detected signals corresponding to another set of non-dominant compound in the NMR spectra of garsubelone B/C and structurally similar compounds: hyperselancins A and B [28], androforin A [31], and hypercohin I [32]. Analysis of the 2D NMR data of garsubelone B/C indicated that these signals correspond to positional isomers of the pyran ring (C-2–O–C-16) in this class of compounds (Scheme 2). Based on the HPLC analysis results of garsubelone B (Fig. S1), it can be inferred that this class of compounds exhibits slow tautomerism, with the dominant isomer having the pyran ring positioned at C-4–O–C-16 (Scheme 2).

**Table 4:** ^1^H and ^13^C NMR data for garcinielliptone F and garsubelone B in CDCl_3_

No.	**Garcinielliptone F**^a^		**Garsubelone B**^b^	
	*δ*_C_, type	*δ*_H_ mult. (*J* in Hz)	*δ*_C_, type	*δ*_H_ mult. (*J* in Hz)
1	83.5, C		83.5, C	
2	188.3, C		188.4, C	
3	114.5, C		114.5, C	
4	170.8, C		170.8, C	
5	56.7, C		56.7, C	
6	39.2, CH_2_	1.89, dd (13.2, 3.2)	39.2, CH_2_	1.89, dd (13.2, 4.0)
		1.39, m		1.40, brt (13.2)
7	43.3, CH	1.44, m	43.4, CH	1.48, m
8	46.8, C		46.8, C	
9	206.3, C		206.3, C	
10	209.0, C		209.0, C	
11	42.5, CH	2.07, m	42.5, CH	2.09, overlap
12 16	20.5, CH_3_	1.11, d (6.4)	20.5, CH_3_	1.11, d (6.6)
13	21.5^c^, CH_3_	1.02, d (6.4)	21.5, CH_3_	1.02, d (6.6)
14	115.4, CH	6.47, d (10.0)	115.4, CH	6.47, d (10.0)
15	123.7, CH	5.34, d (10.0)	123.7, CH	5.34, d (10.0)
16	81.9, C		81.9, C	
17	28.6, CH_3_	1.39, s	28.6, CH_3_	1.39, s
18	28.3, CH_3_	1.43, s	28.4, CH_3_	1.44, s
19	29.0, CH_2_	2.49, dd (14.8, 6.0)	29.0, CH_2_	2.49, dd (14.2, 6.9)
		2.41, dd (14.8, 8.0)		2.41, dd (14.2, 6.9)
20	119.3, CH	5.00, t (7.0)	119.3, CH	5.00, t (6.9)
21	133.3, C		133.8, C	
22	25.7^d^, CH_3_	1.65^e^, s	25.7, CH_3_	1.65, s
23	18.1, CH_3_	1.67, s	18.1, CH_3_	1.67, s
24	26.5, CH_2_	2.09, m	26.5, CH_2_	2.09, overlap
		1.67, m		1.63, overlap
25	122.6, CH	4.95, t (7.2)	122.7, CH	4.96, t (7.3)
26	133.8, C		133.3, C	
27	25.9, CH_3_	1.64, s	25.9, CH_3_	1.64, s
28	17.8^d^, CH_3_	1.54^e^, s	17.8, CH_3_	1.54, s
29	22.9, CH_3_	1.22, s	23.0, CH_3_	1.22, s
30	15.6^c^, CH_3_	1.00, s	15.7, CH_3_	1.00, s

^a^Data from the literature

^b^Data that we acquired

^c,d,e^Exchanged pairs of signals, respectively

**Scheme 3:**
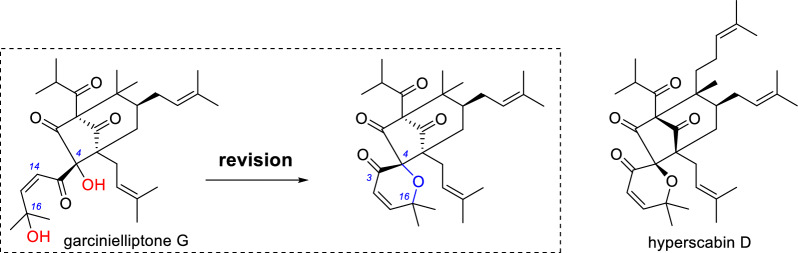
Structural revision of garcinielliptone G

Garcinielliptone G was reported along with garcinielliptone F and was later found to induce apoptosis in acute leukemia cells [29, 30, 33]. However, the structure of garcinielliptone G was also misassigned due to the misuse of MS data. In 2021, hyperscabin D with the same skeleton was reported [34], and its ^1^H and ^13^C NMR data (particularly the signals near the pyran ring) showed excellent agreement with those of garcinielliptone G (Table 5). The IR spectrum of hyperscabin D did not show absorption peak corresponding to hydroxyl groups, nor were any signals of active hydrogen observed in the ^1^H spectrum [34]. The above evidence supports the correct assignment of hyperscabin D and our structural revision of garcinielliptone G (Scheme 3).

**Table 5:** ^1^H and ^13^C NMR data for garcinielliptone G and hyperscabin D in CDCl_3_

No.	**Garcinielliptone G**^a^		**Hyperscabin D**^a,b^	
	*δ*_C_, type	*δ*_H_ mult. (*J* in Hz)	*δ*_C_, type	*δ*_H_ mult. (*J* in Hz)
1	82.8, C		83.7, C	
2	209.1, C		209.7, C	
3	192.4, C		192.6, C	
4	90.2, C		89.8, C	
5	61.0, C		60.8, C	
6	40.2, CH_2_	2.53, m	39.6, CH_2_	2.61, dd (13.8, 4.4)
		1.34, m		1.37, dd (13.8, 10.5)
7	43.1, CH	1.65, m	43.5, CH	1.85, m
8	55.8, C		57.9, C	
9	205.7, C		205.3, C	
10	206.5, C		206.9, C	
11	39.7, CH	2.50, m	39.7, CH	2.50, sept (6.6)
12 16	19.7, CH_3_	1.01, d (6.8)	19.8, CH_3_	1.03, d (6.6)
13	19.6, CH_3_	0.90, d (6.8)	19.7, CH_3_	0.95, d (6.6)
14	122.8, CH	6.01, d (10.8)	123.0, CH	6.02, d (10.5)
15	156.2, CH	6.97, d (10.8)	156.0, CH	6.98, d (10.5)
16	74.1, C		74.2, C	
17	30.0, CH_3_	1.67, s	30.1, CH_3_	1.69, s
18	29.9, CH_3_	1.44, s	29.8, CH_3_	1.45, s
19	27.4, CH_2_	1.60, m	28.0, CH_2_	2.08, m
		2.05, dd (13.2, 8.0)		2.50, m
20	122.5, CH	5.04, t (6.4)	118.6, CH	5.14, t (6.3)
21	133.0, C		133.6, C	
22	25.7, CH_3_	1.71, s	25.9, CH_3_	1.63, s
23	17.8, CH_3_	1.58, s	17.7, CH_3_	1.47, s

^a^Data from the literature

^b^Partial data

**Scheme 4:**
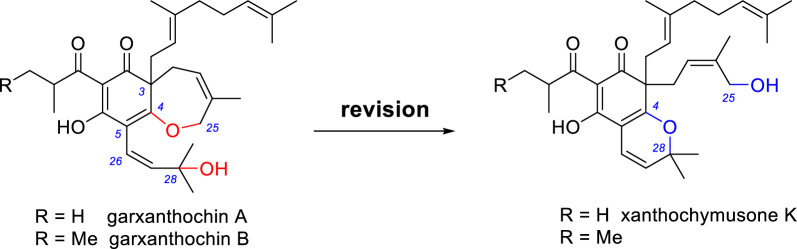
Structural revision of garxanthochins A and B

In 2023, Quan et al. reported two polyprenylated acylphloroglucinols, garxanthochins A and B (Scheme 4), from *G. xanthochymus* [35]. However, the ^1^H and ^13^C NMR data of garxanthochin A are identical to those of xanthochymusone K reported by our group (Table 6) [16]. The chemical shifts of C-25 (*δ*_C_ 61.8) and C-28 (*δ*_C_ 81.5) does not support the linkage of C-4–O–C-25 but support the linkage of C-4–O–C-28. In the ^1^H–^1^H COSY spectrum, a broad singlet at *δ*_H_ 2.22 (hydroxyl hydrogen) shows correlation with *δ*_H_ 3.79 (H-25b) [16], which further confirms our structural reassignment of garxanthochin A. Since garxanthochin B has been assigned the same carbon skeleton as garxanthochin A and their NMR data are similar except for the C-1 acyl substituent, we also revise the structure of garxanthochin B (Scheme 4).

**Table 6:** ^1^H and ^13^C NMR data for garxanthochins A and B and xanthochymusone K in CDCl_3_

No.	**Garxanthochin A**^a^		**Xanthochymusone K**^b^		**Garxanthochin B**^a^	
	*δ*_C_, type	*δ*_H_ mult. (*J* in Hz)	*δ*_C_, type	*δ*_H_ mult. (*J* in Hz)	*δ*_C_, type	*δ*_H_ mult. (*J* in Hz)
1	107.3, C		107.1, C		107.9, C	
2	196.4, C		196.1, C		196.5, C	
3	57.8, C		57.6, C		57.8, C	
4	171.4, C		171.2, C		171.4, C	
5	106.8, C		106.5, C		106.9, C	
6	186.9, C		186.7, C		187.0, C	
7	208.1, C		207.9, C		207.6, C	
8	35.8, CH	3.92, m	35.6, CH	3.95, sept (6.8)	42.1, CH	3.81, dd (13.7, 6.9)
9	19.3, CH_3_	1.12^c^, d (6.8)	19.1, CH_3_	1.12, d (6.8)	16.4, CH_3_	1.10, m
10	18.8, CH_3_	1.07^c^, d (6.8)	18.6, CH_3_	1.10, d (6.8)	26.9, CH_2_	1.33, m
10a					12.0, CH_3_	0.92, t, (7.4)
11	39.1, CH_2_	2.61, dd (13.7, 7.8)	38.9, CH_2_	2.63, dd (13.6, 7.4)	39.0, CH_2_	2.61, dd (13.7, 7.7)
		2.48, d (7.1)		2.51, dd (13.6, 7.4)		2.48, d (7.2)
12 16	117.4, CH	4.81, t (7.2)	117.2, CH	4.83, t (7.4)	117.4, CH	4.80, dd (8.1, 7.0)
13	139.5, C		139.2, C		139.4, C	
14	16.6, CH_3_	1.51, t (3.5)	16.4, CH_3_	1.54, s	16.6, CH_3_	1.51, d (1.9)
15	40.0, CH_2_	1.84, d (6.6)	39.7, CH_2_	1.89, m	40.0, CH_2_	1.83, m
				1.85, m		
16	26.9, CH_2_	1.87, m	26.7, CH_2_	1.89, m	27.1, CH_2_	1.86, m
				1.86, m		
17	124.1, CH	4.96, dd (6.7. 5.4)	123.9, CH	4.98, t (6.8)	124.1, CH	4.95, m
18	131.8, C		131.6, C		131.8, C	
19	25.9, CH_3_	1.61, s	25.6, CH_3_	1.63, s	25.9, CH_3_	1.61, d (0.7)
20	17.8, CH_3_	1.54^d^, s	17.6, CH_3_	1.54, s	17.8, CH_3_	1.54^d^, s
21	35.7, CH_2_	2.92, dd (13.5, 10.4)	35.5, CH_2_	2.94, dd (13.6, 10.2)	35.9, CH_2_	2.92, dd (13.5, 10.4)
		2.50, d (7.1)		2.51, m		2.50, d (6.8)
22	122.1, CH	4.90, dd (10.7, 6.0)	121.8, CH	4.92, dd (10.2, 5.5)	122.0, CH	4.90, dd (9.8, 4.2)
23	138.5, C		138.4, C		138.6, C	
24	22.3, CH_3_	1.66, s	22.1, CH_3_	1.68, s	22.3, CH_3_	1.65, d (1.2)
25	**61.8**, CH_2_	4.29, d (12.0)	61.6, CH_2_	4.30, d (12.0)	**61.8**, CH_2_	4.30, dd (14.7, 12.2)
		3.78, d (12.0)		3.79, brd (12.0)		3.77, m
26	114.8, CH	6.45, d (10.0)	114.6, CH	6.47, d (10.0)	114.8, CH	6.45, d (10.1)
27	123.8, CH	5.36, d (10.1)	123.5, CH	5.38, d (10.0)	123.8, CH	5.35, d (10.1)
28	**81.5**, C		81.3, C		**81.5**, C	
29	29.0, CH_3_	1.46, s	28.8, CH_3_	1.48, s	29.0, CH_3_	1.46, s
30	28.9, CH_3_	1.45, s	28.7, CH_3_	1.47, s	28.9, CH_3_	1.45, s

^a^Data from the literature

^b^Data that we acquired

^c^Exchanged signals

^d^Revised data based on the spectra provided by the original authors

**Scheme 5:**
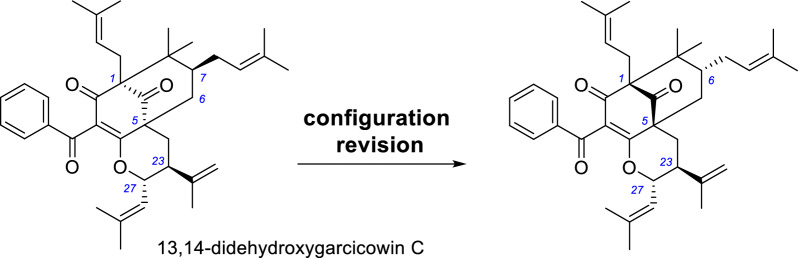
Structural revision of 13,14-didehydroxygarcicowin C

Since we revised the relative configuration of garcicowins C and D in 2022 [13], we have further noticed that their derivative, 13,14-didehydroxygarcicowin C [36], also exhibits the same configurational error. As shown in Table 7, the ^1^H and ^13^C NMR data of the core carbon skeleton of 13,14-didehydroxygarcicowin C shows excellent agreement (particularly around the C-30 and C-34 stereocenters) with those of xanthochymusone H, whose structure was confirmed by single-crystal X-ray diffraction data [13]. So we firstly revise the relative configuration of C-23 and C-27 of 13,14-didehydroxygarcicowin C. Furthermore, the opposite optical rotations of 13,14-didehydroxygarcicowin C and xanthochymusone H ([*α*]_D_ = –68.6 and + 60, respectively) and almost opposite experimental CD data indicate that their absolute configurations are opposite [*λ*_max_ (Δ*ε*) 223 (+ 5.2), 267 (− 8.5), 311 (+ 2.1) nm for 13,14-didehydroxygarcicowin C; *λ*_max_ (Δ*ε*) 224 (− 8.8), 258 (+ 7.2), 302 (− 0.25) nm for xanthochymusone H] [13, 36]. Ultimately, we reassign the absolute configuration of 13,14-didehydroxygarcicowin C as shown (Scheme 5).

**Table 7:** Partial ^1^H and ^13^C NMR data for 13,14-didehydroxygarcicowin C and xanthochymusone H

No.	**13,14-didehydroxygarcicowin C**^a^		**Xanthochymusone H**^b^	
	*δ*_C_, type	*δ*_H_ mult. (*J* in Hz)	*δ*_C_, type	*δ*_H_ mult. (*J* in Hz)
1	69.2 C		70.7, C	
2	193.8, C		196.1, C	
3	129.1, C		124.2, C	
4	170.8, C		173.9, C	
5	48.1, C		49.5, C	
6	38.1, CH	2.60, d (14.5)	38.8, CH_2_	2.63, m
		1.89, dd (14.5, 7.5)		1.95, dd (14.3, 7.2)
7	46.4, CH	1.51, m	47.6, CH	1.58, overlap
8	46.7, C		47.8, C	
9	209.0, C		209.6, C	
22	33.2, CH_2_	2.32, t (14.0)	34.5, CH_2_	2.34, t (14.1)
		1.75, dd (14.0, 2.5)		1.74, dd (14.1, 2.8)
23	42.8, CH	2.46, m	44.2, CH	2.52, m
24	143.5, C		145.5, C	
25	113.9, CH_2_	4.84, s	114.5, CH_2_	4.85, s
		4.80, s		4.84, s
26	20.3, CH_3_	1.66, s	20.7, CH_3_	1.67, s
27	79.7, CH	4.20, t (9.0)	81.6, CH	4.33, dd (10.6, 8.9)
28	121.4, CH	5.01, brd (9.0)	124.2, CH	5.04, d (8.9)
29	141.7, C		143.3, C	
30	25.7, CH_3_	1.61, s	25.9, CH_3_	1.62, s
31	17.8, CH_3_	1.10, s	18.1, CH_3_	1.14, s
37	26.8, CH_3_	1.00, s	27.2, CH_3_	1.01, s
38	22.3, CH_3_	1.18, s	22.8, CH_3_	1.16, s

^*a*^Data from the literature, recorded in CDCl_3_

^*b*^Data from the literature, recorded in methanol-*d*_4_

The antiproliferative activities of all the isolates against two human hepatocellular carcinoma cell lines Huh-7 and Hep G2 through CCK-8 assay were preliminary evaluated [37]. Sorafenib was chosen as the positive control (IC_50_ 9.7 and 16.6 µM, respectively). Compound **1** exhibited moderate cytotoxic activities against Hep G2 cells with IC_50_ value 7.3 µM, while other compounds did not show obvious activity (IC_50_ > 50 µM).

In conclusion, we have isolated six new polyprenylated acylphloroglucinols (**1**–**6**) and two known analogues from the fruits of *G. xanthochymus* and *G. subelliptica*. Compound **1** exhibits moderate antiproliferative activity against HepG2 cell lines. Furthermore, the structures of seven polyprenylated acylphloroglucinols (hyperselancins A and B, garcinielliptones F and G, garxanthochins A and B, and 13,14-didehydroxygarcicowin C) are revised by analysis of NMR and other spectroscopic data. Our structural confirmation work has cleared the obstacles for subsequent research on these natural products.

## Experimental section

### General experimental procedures

Optical rotations were measured on a Jasco P-1020 polarimeter. UV spectra were recorded on a Shimadzu UV-2401PC spectrometer. IR spectra were recorded on a Bruker FT-IR Tensor-27 infrared spectrophotometer with KBr disks. 1D and 2D NMR spectra were recorded on a Bruker DRX-600 spectrometer using TMS as an internal standard. Unless otherwise specified, chemical shifts (*δ*) are expressed in ppm with reference to the solvent signals. ESIMS and HREIMS data were acquired on Waters Xevo TQS and Waters AutoSpec Premier P776 mass spectrometers, respectively. Semi-preparative HPLC was performed on an Agilent 1100 HPLC with a Zorbarx SB-C_18_ (9.4 × 250 mm) column. Silica gel (200–300 mesh, Qingdao Marine Chemical Co., Ltd., Qingdao, People’s Republic of China) were used for column chromatography. Fractions were monitored by TLC (GF 254, Qingdao Marine Chemical Co., Ltd.), and spots were visualized by heating silica gel plates immersed in H_2_SO_4_ in EtOH.

### Plant material

James Stribling and Louise King collected the fruits of *G. xanthochymus* from the Fruit and Spice Park (Miami, FL) in February 2020. Prof. Xing-Wei Yang and his students collected the fruits of *G. subelliptica* from the treelawn nearby the Shenzhen campus of Sun Yat-sen University in August 2022.

### Extraction and isolation

The dried fruits of* G. xanthochymus* (1.2 kg) were extracted twice with MeOH (5 L each time) at room temperature for 24 h to yield a crude extract (680 g) after evaporation in vacuo. The residue was suspended in H_2_O and partitioned with EtOAc and H_2_O to yield the EtOAc fraction (120 g). This fraction was subjected to column chromatography over silica gel eluted with petroleum ether–EtOAc in gradients (20:1, 10:1, 6:1, 5:1, 5:2, 1:1, and 0:1) to obtain ten fractions (Fr. A–J). A portion of Fr. D (5 g) was separated by semipreparative HPLC eluting with MeOH–H_2_O (88:12) to yield four subfractions (Fr. D1-D4). Fr. D4 (174 mg) was further purified via preparative TLC and semipreparative HPLC to afford compounds **2** (16 mg) and **3** (15 mg). Fr. E (11.8 g) was chromatographed on a silica gel column, eluting with petroleum ether–EtOAc (3:1 to 0:1), to gather Fr. E1–E3. Using semipreparative HPLC (MeCN–H_2_O, 65:35), Fr. E3.4 (548 mg) afforded compound **1** (8 mg).

The dried fruits of *G. subelliptica* (5.3 kg) were extracted three times with MeOH (6–8 L each time) at room temperature for 24 h to yield a crude extract after evaporation in vacuo. The residue was suspended in H_2_O and partitioned with EtOAc and H_2_O to yield the EtOAc fraction (211 g). This fraction was subjected to column chromatography over silica gel eluting with petroleum ether–EtOAc in gradients (95:1, 9:1, 4:1, 1:1, 1:2, and 0:1) and industrial MeOH to obtain six fractions (Fr. A–F). Fr. A (14.4 g) was fractionated by silica gel column chromatography using petroleum ether–EtOAc (200:1 to 100:1) as eluents to provide three subfractions (Fr. A1–A3). Fr. A2 (1.5 g) was purified by semi-preparative HPLC (MeCN–H_2_O, 99:1 to 100:0) to produce compounds **5** (9 mg), **6** (12 mg), and **8** (3 mg). A portion (200 mg) of A3 (6.7 g) was purified by semipreparative HPLC (MeCN–H_2_O, 95:5 to 100:0) to obtain compounds **4** (10 mg) and **7** (16 mg).

*Xanthochymusone N (****1****)*: yellow gum; $[\alpha]^{25}_{\text{D}}$ –16 (*c* 0.1, MeOH); UV (MeOH)* λ*_max_ (log *ε*) 232 (4.07), 293 (3.82), 327 (3.78) nm; IR (KBr) *v*_max_ 3400, 2918, 2850, 1724, 1621, 1467, 1377, 1261, 1095, 1032, 802, 667 cm^−1^; ^1^H and ^13^C NMR data, see Table 1; negative ESIMS *m/z* 635 [M – H]^−^; HRESIMS *m/z* 635.3586 (cacld for C_38_H_51_O_8_, 635.3584).

*Xanthochymusone O (****2****)*: yellow gum; $[\alpha]^{25}_{\text{D}}$ –21 (*c* 0.1, MeOH); UV (MeOH)* λ*_max_ (log *ε*) 206 (4.25), 234 (3.91), 293 (3.56), 333 (3.53) nm; IR (KBr) *v*_max_ 3427, 2968, 2928, 2857, 1726, 1624, 1474, 1394, 1380, 1290, 1178, 889 cm^−1^; ^1^H and ^13^C NMR data, see Table 1; HRESIMS *m/z* 601.3522 [M + H]^+^ (cacld for C_38_H_49_O_6_, 601.3524).

*(–)-Garciyunnanin L (3)*: yellow gum; $[\alpha]^{25}_{\text{D}}$ –21 (*c* 0.1, MeOH); CD (0.0002 M, MeOH) *λ*_max_ (Δ*ε*) 211 (− 13.5), 230 (+ 1.9), 250 (− 1.2), 285 (− 4.4), 305 (+ 3.5), 330 (− 0.8) nm.

*Garsubelone C* (***4***): colorless gum; $[\alpha]^{25}_{\text{D}}$ + 6 (*c* 0.06, MeOH); UV (MeOH)* λ*_max_ (log *ε*) 204 (3.97), 258 (3.69), 317 (3.38) nm; IR (KBr) *v*_max_ 2972, 2928, 1727, 1639, 1587, 1453, 1413, 1370, 1336, 1115 cm^−1^; CD (0.0004 M, MeOH) *λ*_max_ (Δ*ε*) 202 (− 8.0), 219 (+ 0.7), 239 (+ 0.7), 265 (− 2.2), 293 (− 1.9), 321 (+ 5.0), 347 (− 0.5) nm; ^1^H and ^13^C NMR data, see Table 2; positive ESIMS *m/z* 519 [M + K]^+^; HRESIMS *m/z* 503.3134 (cacld for C_31_H_44_O_4_Na, 503.3137).

*Garsubelone D* (***5***): red gum; $[\alpha]^{25}_{\text{D}}$ + 220 (*c* 0.05, MeOH); UV (MeOH)* λ*_max_ (log *ε*) 209 (4.13), 238 (3.89), 332 (3.74) nm; IR (KBr) *v*_max_ 3424, 2975, 2927, 1659, 1526, 1447, 1382, 1179, 1143, 1107, 891, 802, 745 cm^−1^; ^1^H and ^13^C NMR data, see Table 5; positive ESIMS *m/z* 489 [M + Na]^+^; HRESIMS *m/z* 489.2978 (cacld for C_30_H_42_O_4_Na, 489.2981).

*Garsubelone E* (***6***): red gum; $[\alpha]^{25}_{\text{D}}$ + 36 (*c* 0.05, MeOH); UV (MeOH)* λ*_max_ (log *ε*) 207 (4.04), 265 (4.05), 354 (3.54) nm; IR (KBr) *v*_max_ 3430, 2973, 2926, 1655, 1526, 1461, 1361, 1196, 1142, 1103, 887, 714 cm^−1^; ^1^H and ^13^C NMR data, see Table 5; positive ESIMS *m/z* 505 [M + K]^+^; HRESIMS *m/z* 489.2976 (cacld for C_30_H_42_O_4_Na, 489.2981).

### Cytotoxicity assay

Two human hepatocellular carcinoma cell lines (Huh-7 and Hep G2) were cultured in DMEM containing 10% FBS at 37 °C with 5% CO_2_. Cells (Huh-7 and Hep G2) were seeded on 96-well plates with 10,000 cells per well and incubated for 24 h. All isolated compounds were added with a serial dilution (50, 25, 12.5, 6.25, 3.125 µM) and cultivated in the cell incubator for another 24 h. 10 µL of the Cell Counting Kit-8 (Biosharp, Shanghai, China) was added to the medium and incubated for 2–4 h, absorbance was measured by Multiskan GO microplate reader at a wavelength of 450 nm. Sorafenib (Solarbio, Shanghai, China) was used as a positive control. The half-maximal inhibitory concentration (IC_50_) value was measured and calculated by GraphPad Prism 8 software.

## Supplementary Information

Additional file1 Transformation between 7 and 7a detected by HPLC (Fig. S1), Original MS and NMR spectra of compounds **1**–**6** (Fig. S2–S29). (PDF 2862 KB)

## Data Availability

All data generated or analyzed during this study are included in this published article and its supplementary information files.
