# Supplementary material for: Polyprenylated acylphloroglucinols from Garcinia species and structural revision of seven analogues
Source: Nat Prod Bioprospect. 2025 May 26;15(1):34. doi: 10.1007/s13659-025-00519-6 (PMC12106175; doi:10.1007/s13659-025-00519-6)
Supplement: Supplementary file 1 — Additional file1 Transformation between 7 and 7a detected by HPLC (Fig. S1), Original MS and NMR spectra of compounds 1–6 (Fig. S2–S29). (PDF 2862 KB) [file 13659_2025_519_MOESM1_ESM.pdf]

Polyprenylated acylphloroglucinols from *Garcinia* species and structural revision of seven analogues

Yong-Ge Fu,[1†] Yi-Qi Huang,[1†] Zhi-Hong Xu,[1] Xia Liu,[2,*] and Xing-Wei Yang[1,*]

[1] School of Pharmaceutical Sciences (Shenzhen), Sun Yat-sen University, Shenzhen 518107, People's Republic of China

[2] Department of Pharmacy, Chongqing Traditional Chinese Medicine Hospital, Chongqing, 400021, China

[†] Yong-Ge Fu and Yi-Qi Huang have authors contributed equally

Supplementary Information

Table of Contents

Transformation between **7** and **7a** detected by HPLC (Page S2)

The original MS and NMR spectra of compound **1** (Page S2–S9)

The original MS and NMR spectra of compound **2** (Page S10–S13)

The original ECD and NMR spectra of compound **3** (Page S13–S14)

The original MS and NMR spectra of compound **4** (Page S15–S19)

The original MS and NMR spectra of compound **5** (Page S20–S24)

The original MS and NMR spectra of compound **6** (Page S25–S29)

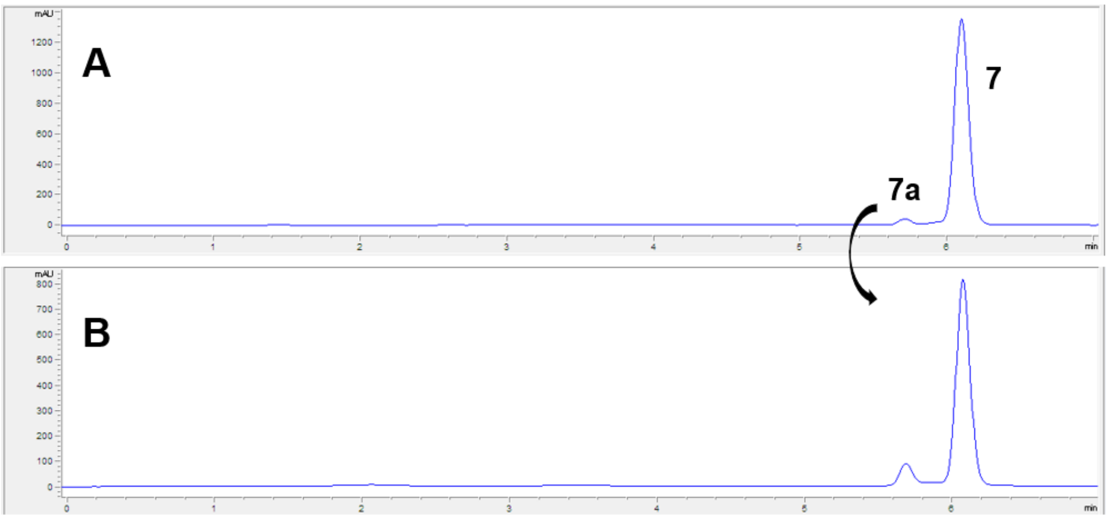

Figure S1. Transformation between **7** and **7a**. A: HPLC isolation chromatogram of compounds **7** and **7a**. B: Immediately analysis chromatogram of the collected sample of **7a**. Chromatographic conditions: Agilent 1100 HPLC with a Zorbax SB-C18 (4.6 × 250 mm) column; flow: 100% MeCN, 1.0 mL/min; temperature: 35 °C; wavelength: 254 nm.

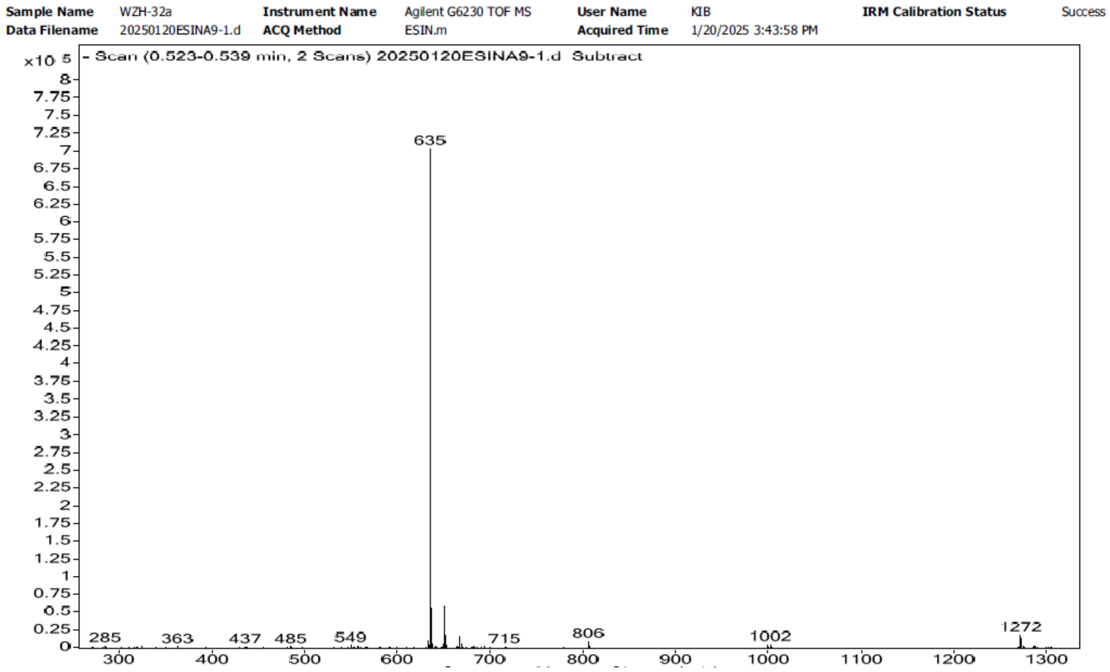

Figure S2. ESIMS of xanthochymusone N (**1**).

Qualitative Analysis Report

**Data Filename**	20250120ESINA9-1.d	**Sample Name**	WZH-32a
**Sample Type**	Sample	**Position**	Vial 81
**Instrument Name**	Agilent G6230 TOF MS	**User Name**	KIB
**Acq Method**	ESIN.m	**Acquired Time**	1/20/2025 3:43:58 PM
**IRM Calibration Status**	Success	**DA Method**	ESI.m
**Comment**			

Sample Group **Info.**

Acquisition SW Version 6200 series TOF/6500 series Q-TOF B.05.01 (B5125.2)

User Spectra

Fragmentor Voltage	Collision Energy	Ionization Mode
150	0	ESI

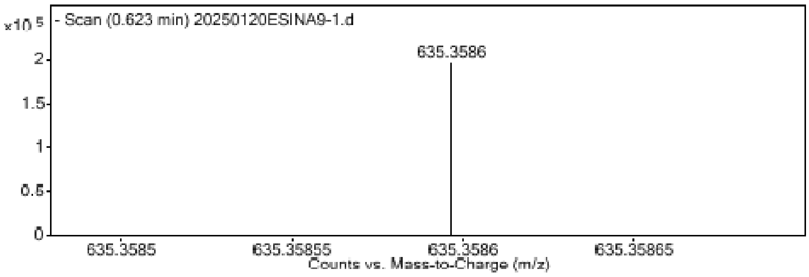

Peak List

m/z	z	Abund	Formula	Ion
112.9856	1	44480.69		
198.7984		19655.11		
635.3586	1	197116.53	C38 H51 O8	M-
636.3614	1	70251.67	C38 H51 O8	M-
715.2822	1	60838.93		
716.2855	1	17500.5		
717.2821	1	63172.03		
749.3492	1	30540.11		
1033.9881	1	232712.2		
1034.989	1	34812.7		

Formula Calculator Element Limits

Element	Min	Max
C	0	200
H	0	400
O	4	10

Formula Calculator Results

Formula	CalculatedMass	Mz	Diff.(mDa)	Diff. (ppm)	DBE
C38 H51 O8	635.3584	635.3586	-0.2	0.3	13.5

--- End Of Report ---

Figure S3. HRESIMS of xanthochymusone N (**1**).

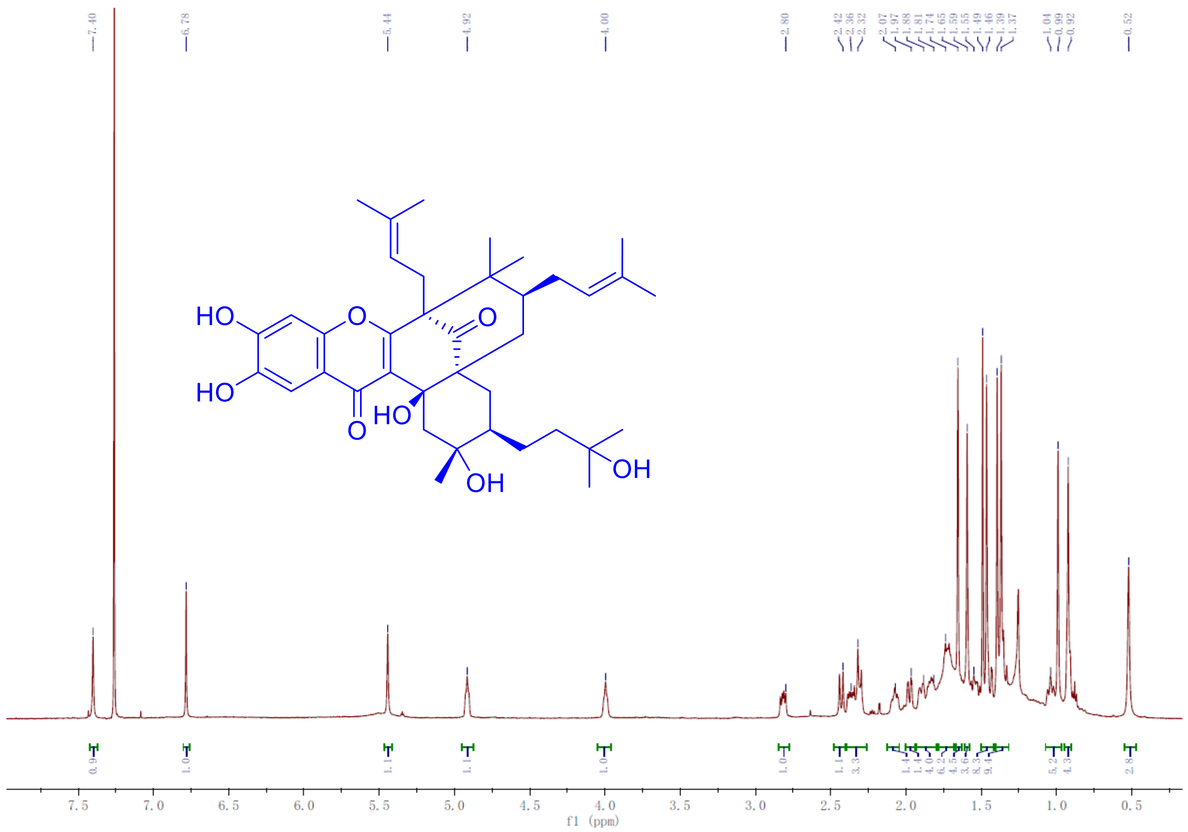

Figure S4. ^{1}H NMR (600 MHz) spectrum of **1** in $CDCl_3$.

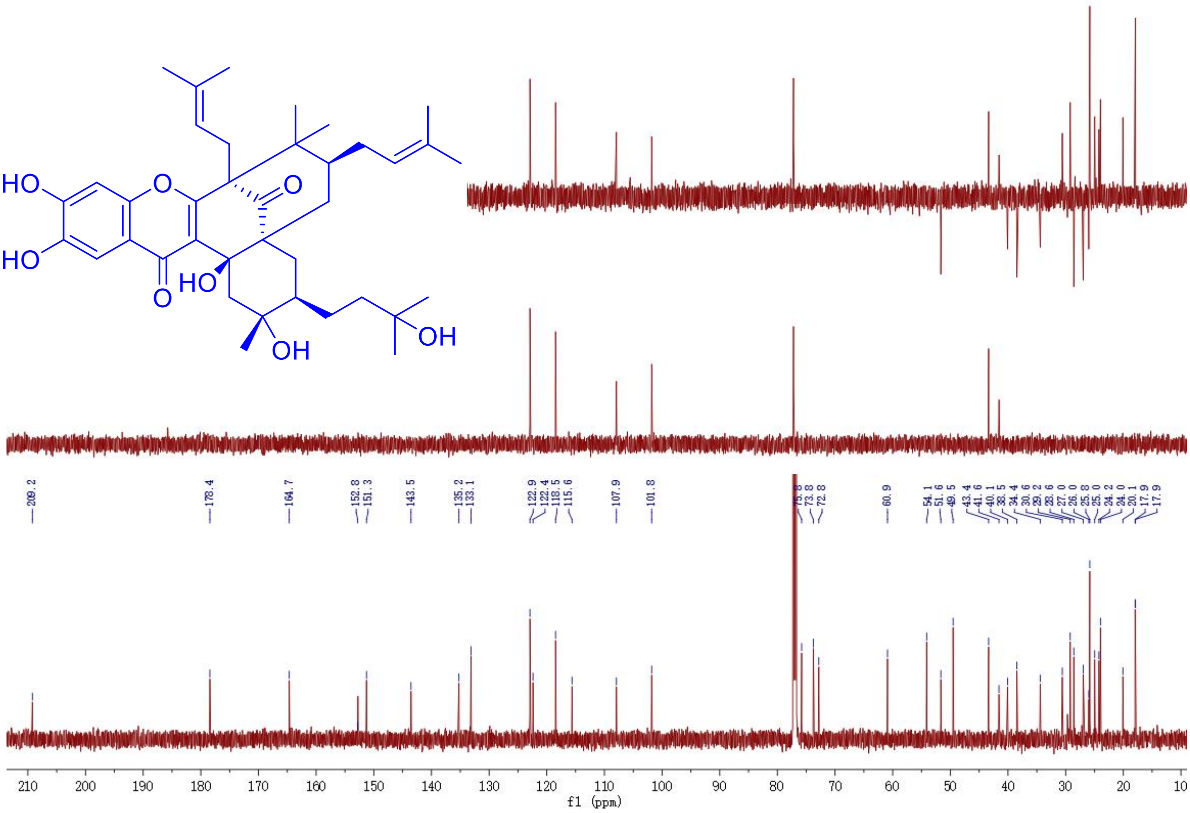

Figure S5. ^{13}C (150 MHz) and DEPT spectra of **1** in $CDCl_3$.

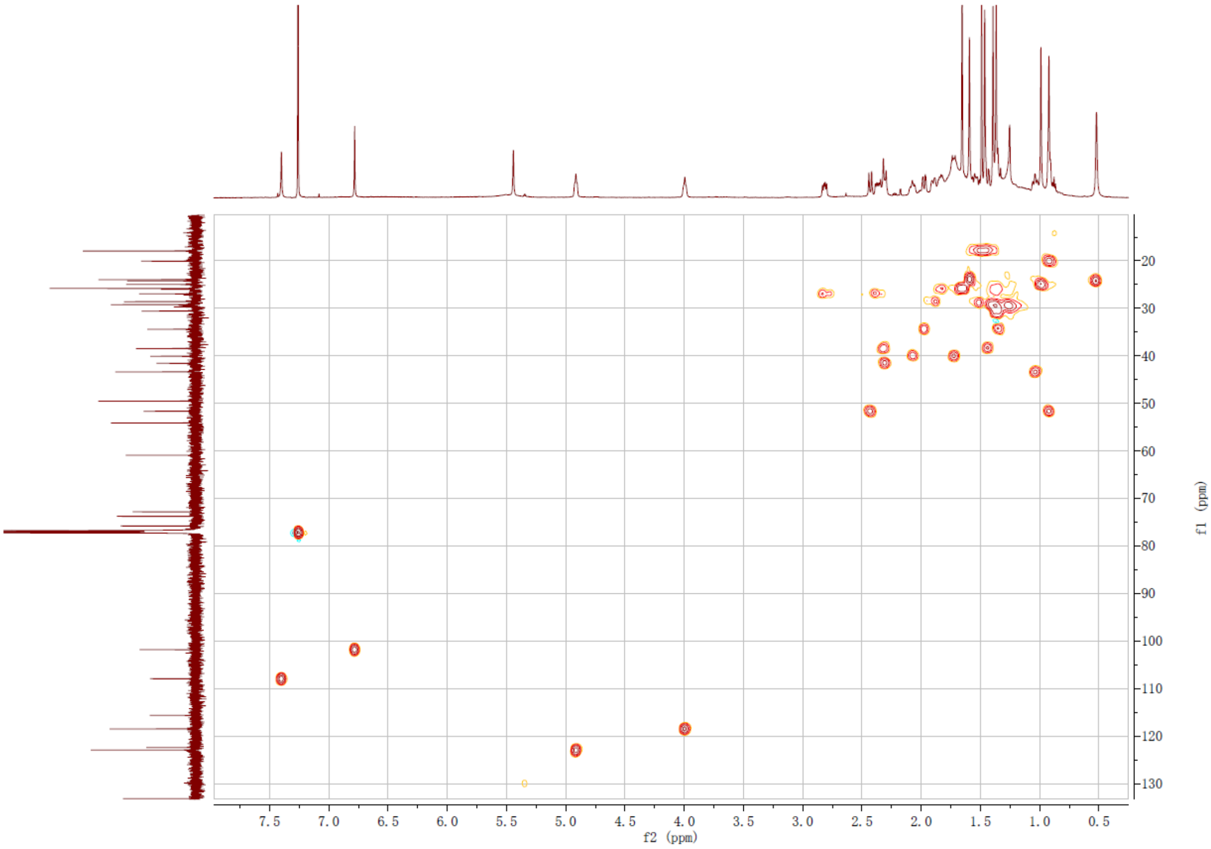

Figure S6. HSQC spectrum of **1** in $CDCl_3$.

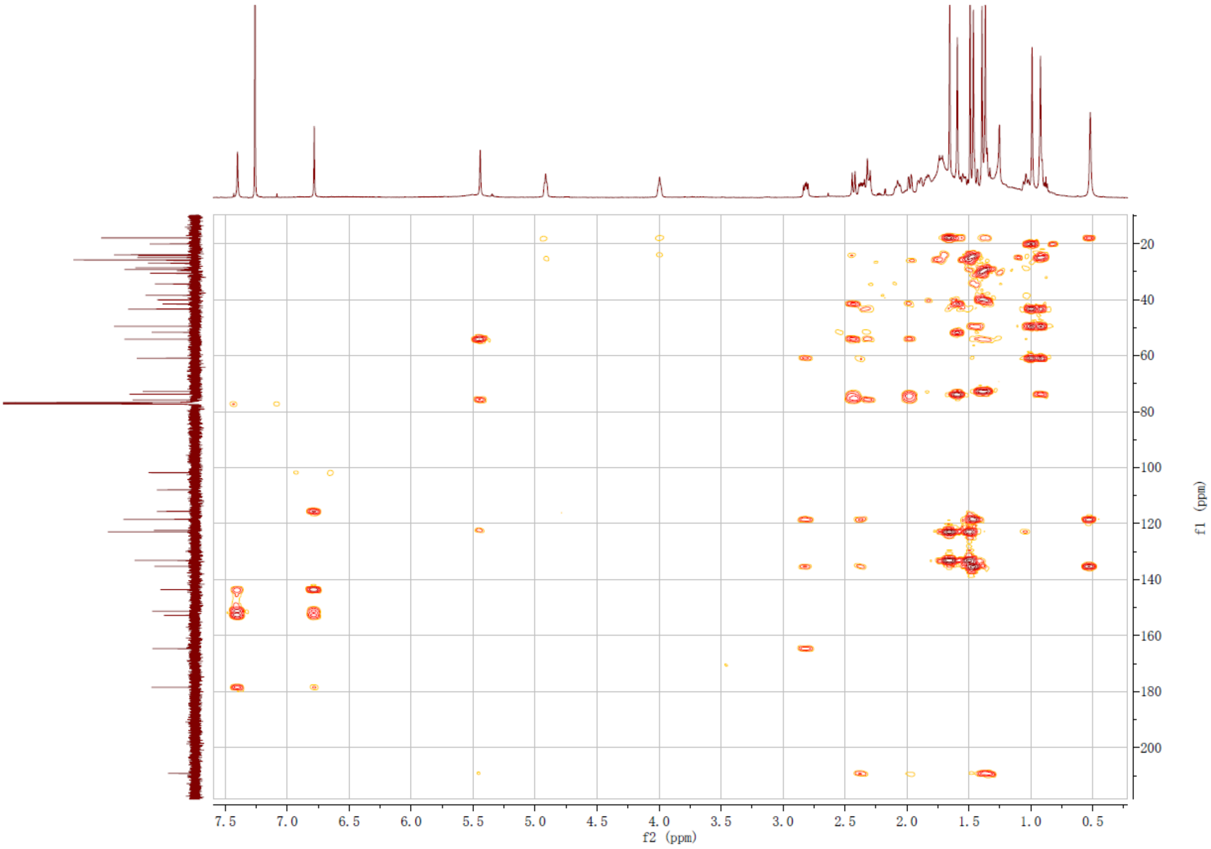

Figure S7. HMBC spectrum of **1** in $CDCl_3$.

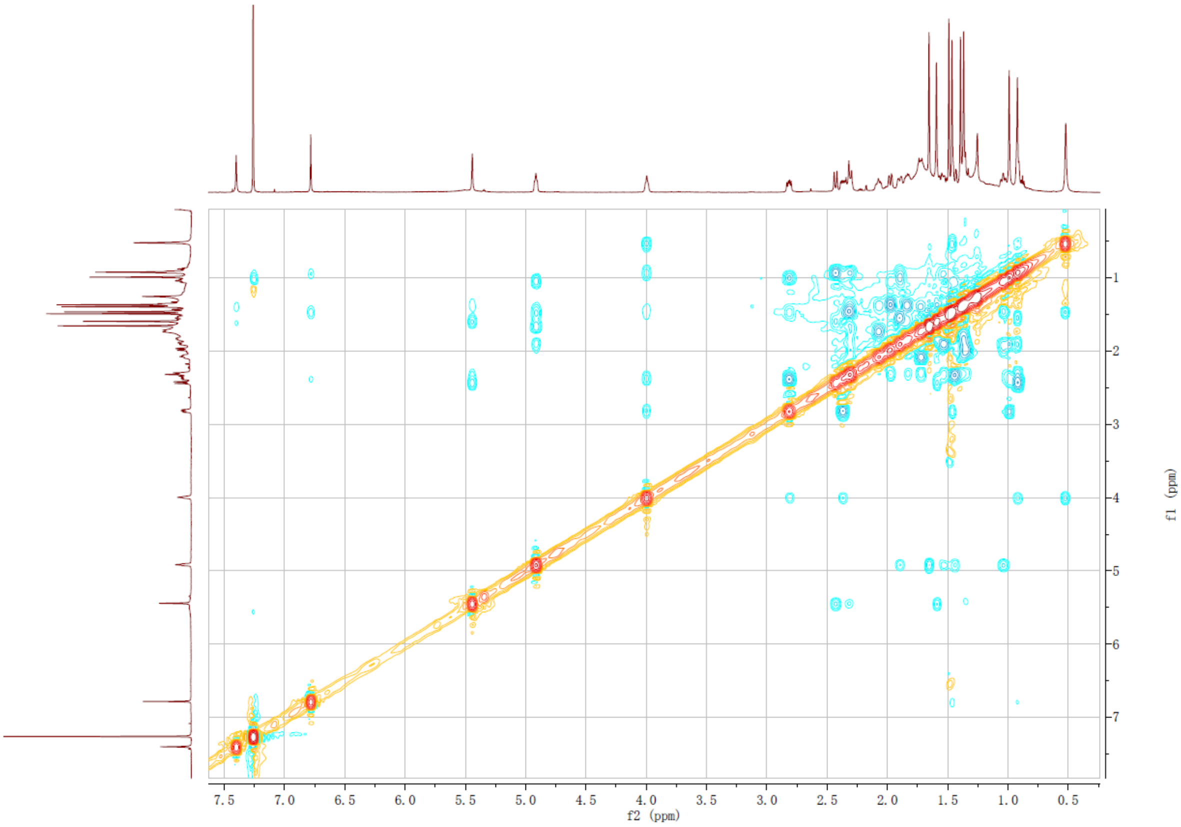

Figure S8. ROESY spectrum of **1** in $CDCl_3$.

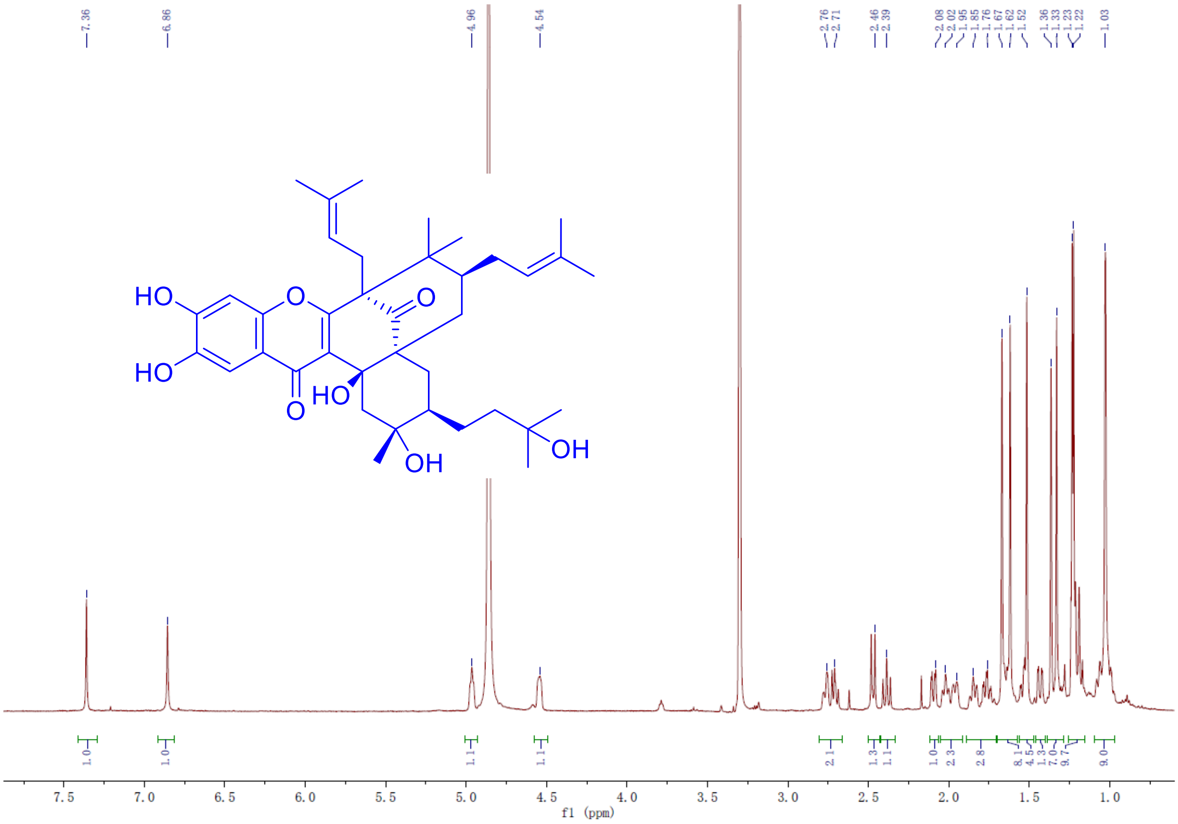

Figure S9. ^{1}H NMR (600 MHz) spectrum of **1** in methanol-d_4.

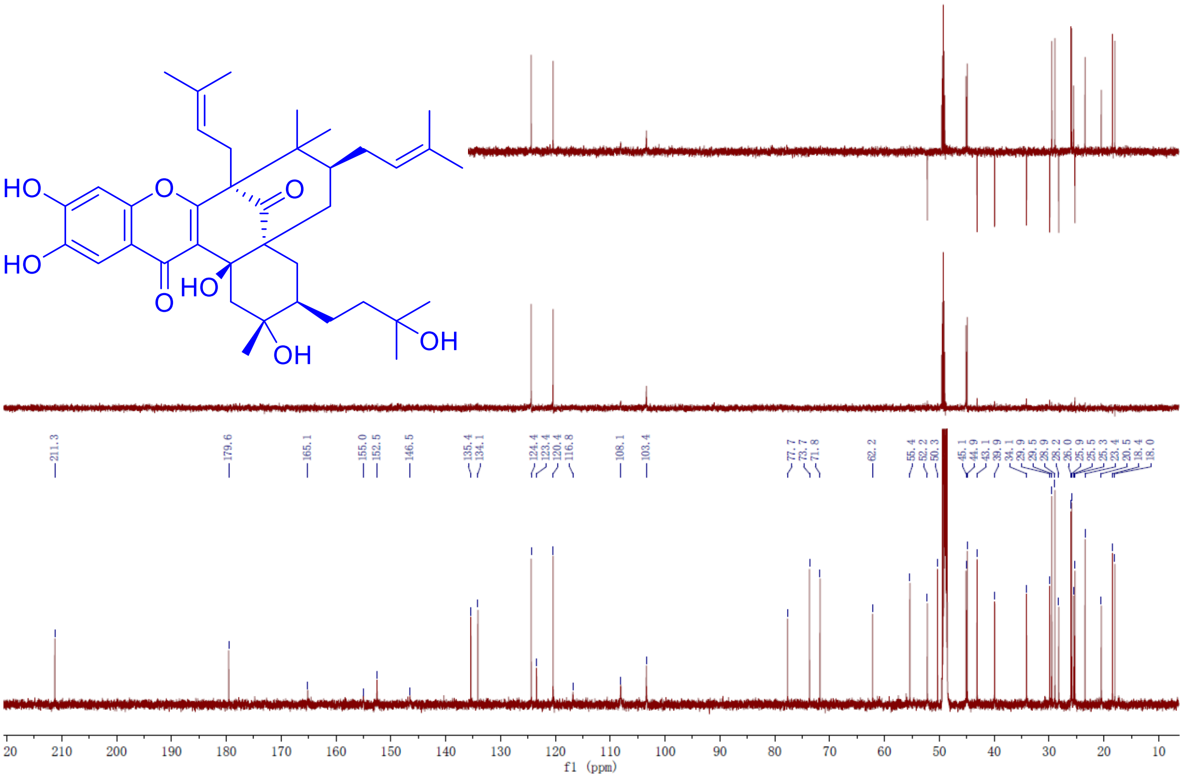

Figure S10. ^{13}C (150 MHz) and DEPT spectra of **1** in methanol-d_4.

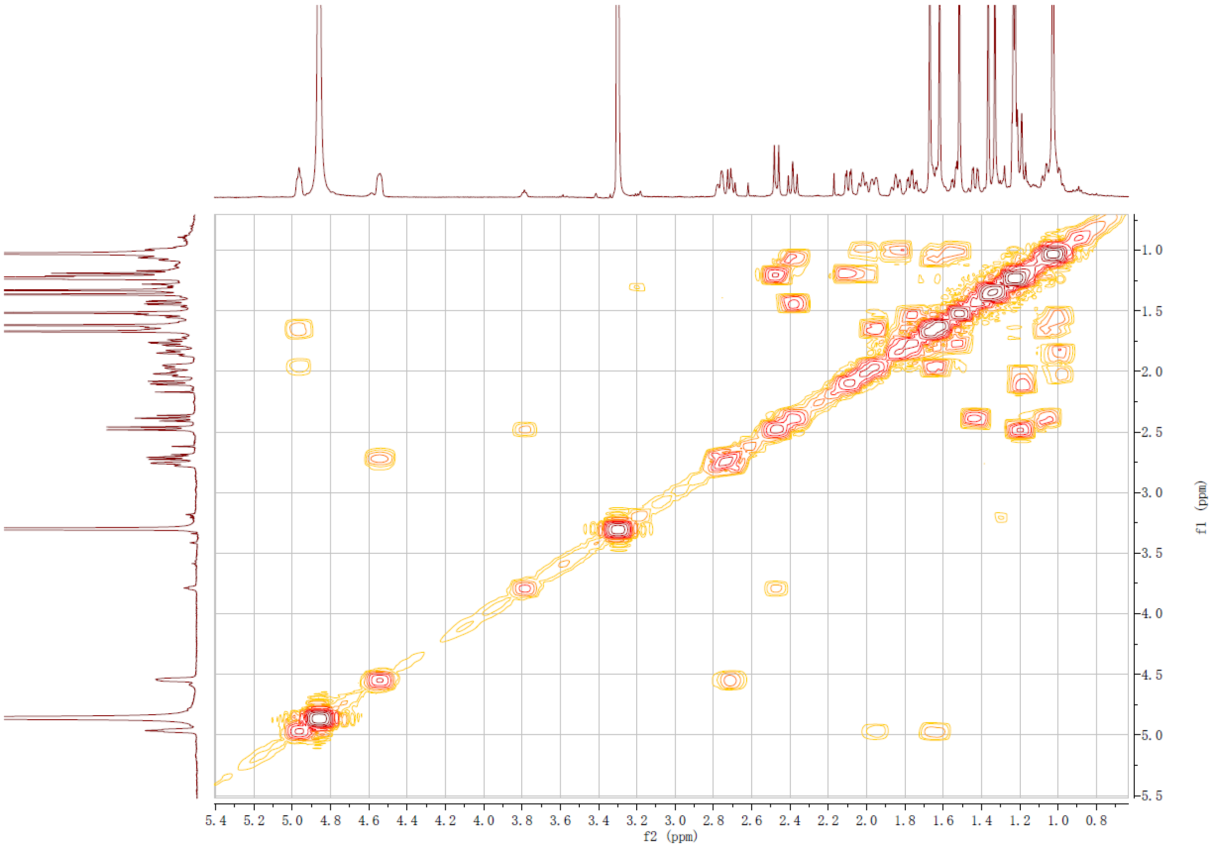

Figure S11. ^{1}H-^{1}H COSY spectrum of **1** in methanol-d_4.

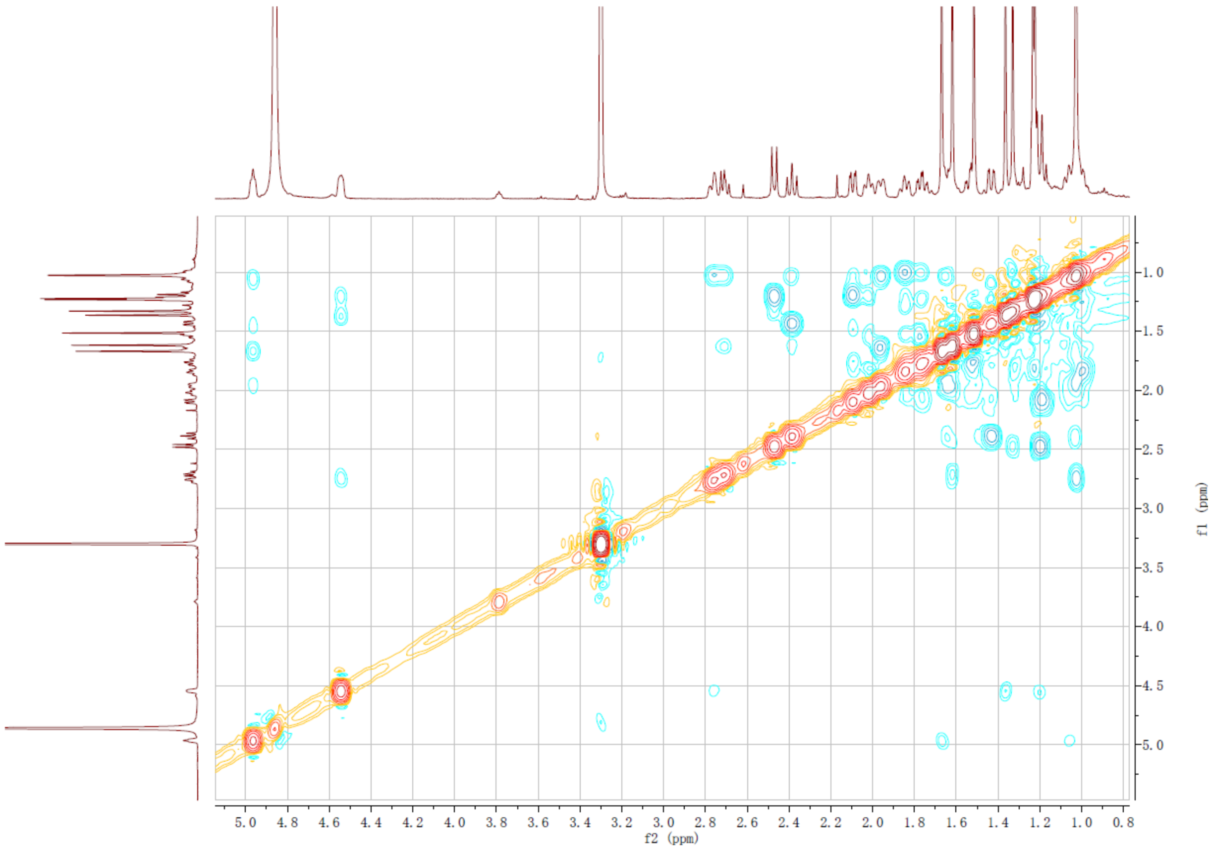

Figure S12. ROESY spectrum of **1** in methanol-d_4.

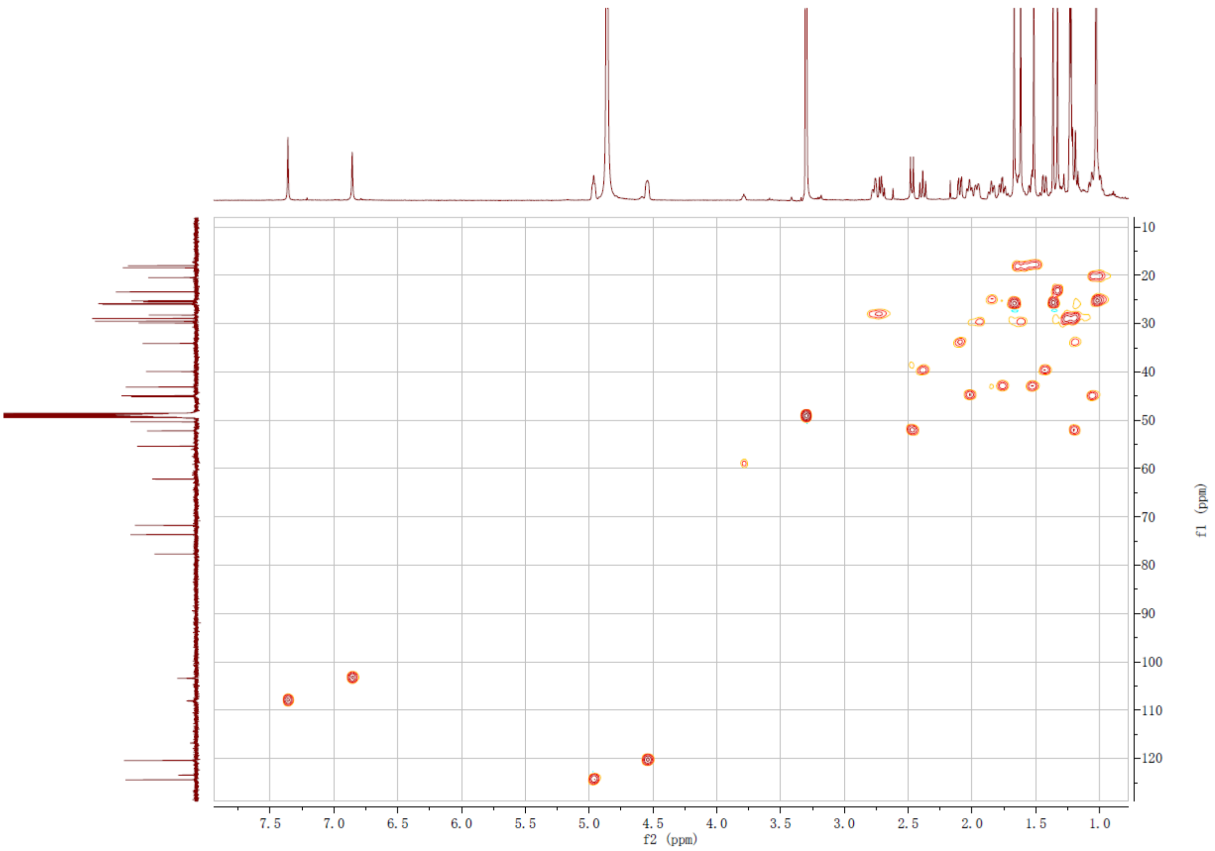

Figure S13. HSQC spectrum of **1** in methanol-d_4.

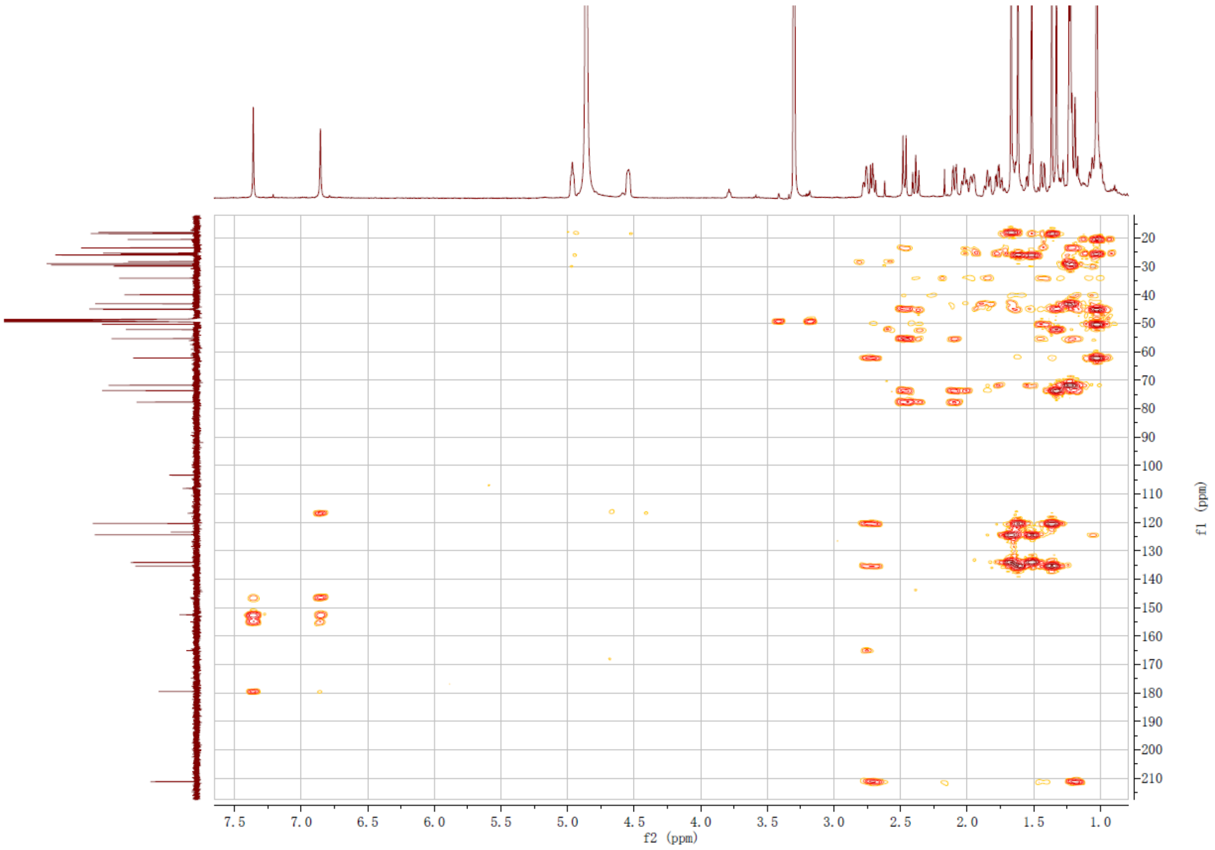

Figure S14. HMBC spectrum of **1** in methanol-d_4.

Qualitative Analysis Report

**Data Filename**	WLy-269-2.d	**Sample Name**	WLy-269-2
**Sample Type**	Sample	**Position**	P1-B1
**Instrument Name**	Instrument 1	**User Name**	
**Acq Method**	s.m	**Acquired Time**	3/17/2023 11:01:37 AM
**IRM Calibration Status**	Success	**DA Method**	PCDL.m
**Comment**			

Sample Group **Info.**

Acquisition SW 6200 series TOF/6500 series

Version Q-TOF B.05.01 (B5125.2)

User Spectra

Fragmentor Voltage	Collision Energy	Ionization Mode
135	0	ESI

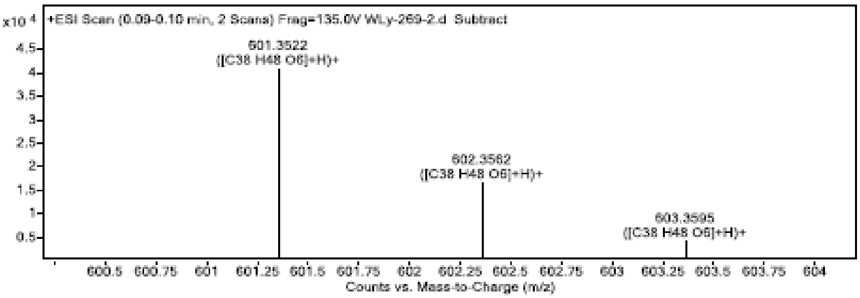

Peak List

m/z	z	Abund	Formula	Ion
98.984	1	2453.88		
274.2735	1	1950.93		
318.3006	1	2239.59		
583.3421	1	21188.43		
584.346	1	9176.57		
585.3484	1	2232.43		
599.3378	1	1455.29		
601.3522	1	40857.4	C38 H48 O6	(M+H)+
602.3562	1	16590.24	C38 H48 O6	(M+H)+
603.3595	1	4141.96	C38 H48 O6	(M+H)+

Formula Calculator Element Limits

Element	Min	Max
C	3	60
H	0	120
O	0	20
N	0	3

Formula Calculator Results

Formula	CalculatedMass	CalculatedMz	Mz	Diff. (mDa)	Diff. (ppm)	DBE
C38 H48 O6	600.3451	601.3524	601.3522	0.20	0.33	15.0000

--- End Of Report ---

Figure S15. HRESIMS of xanthochymusone O (**2**).

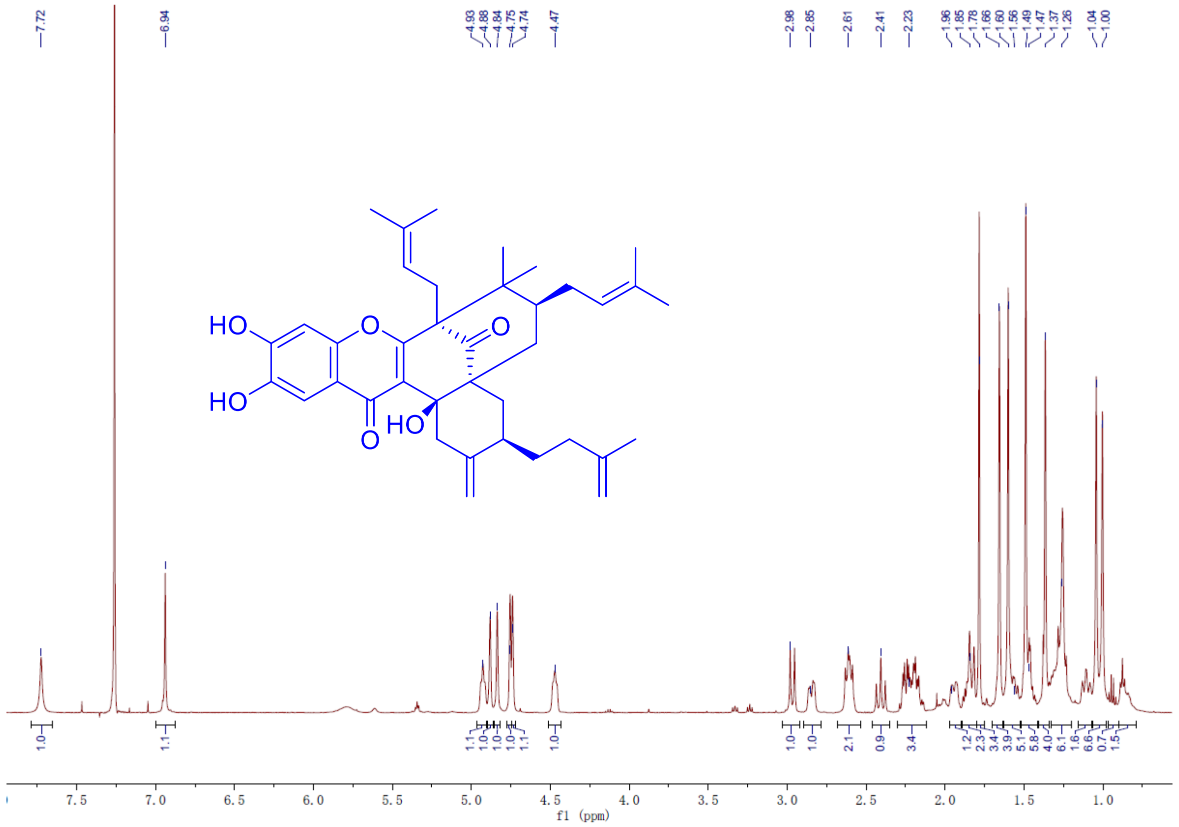

Figure S16. ^{1}H NMR (500 MHz) spectrum of **2** in $CDCl_3$.

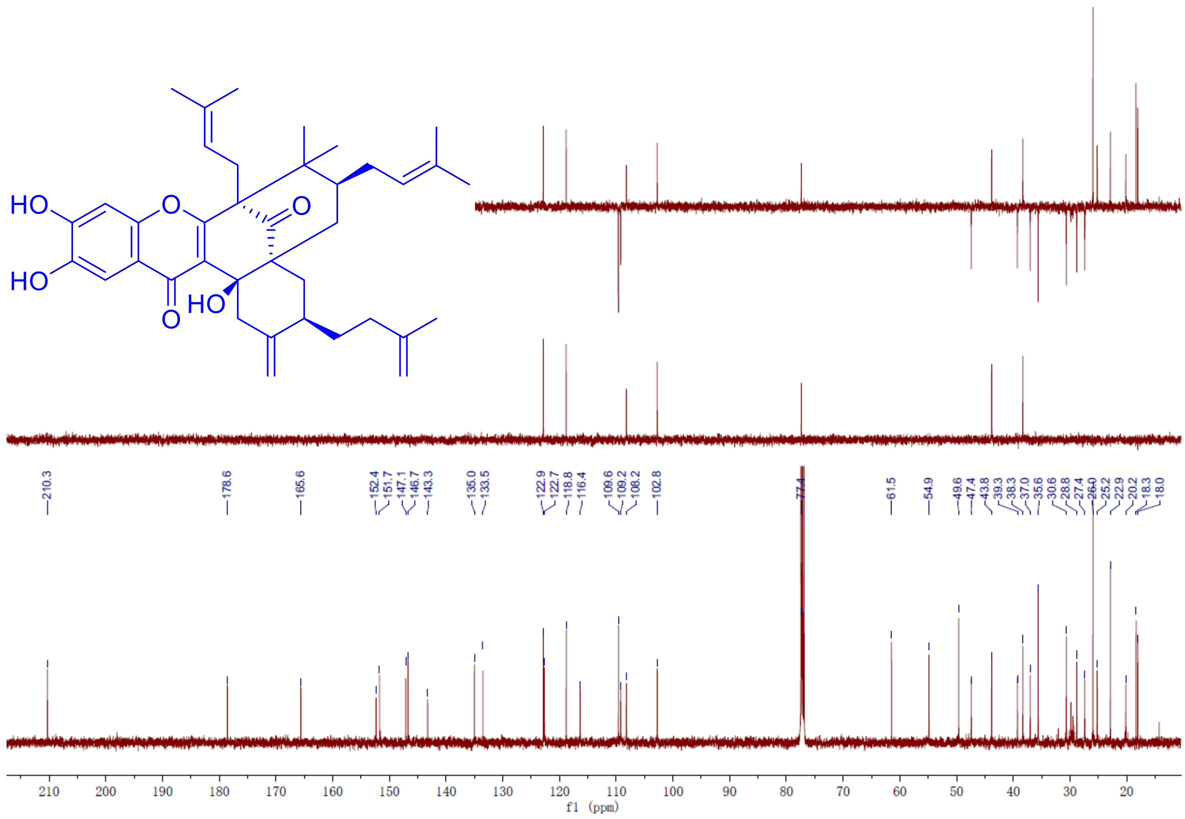

Figure S17. ^{13}C (125 MHz) and DEPT spectra of **2** in $CDCl_3$.

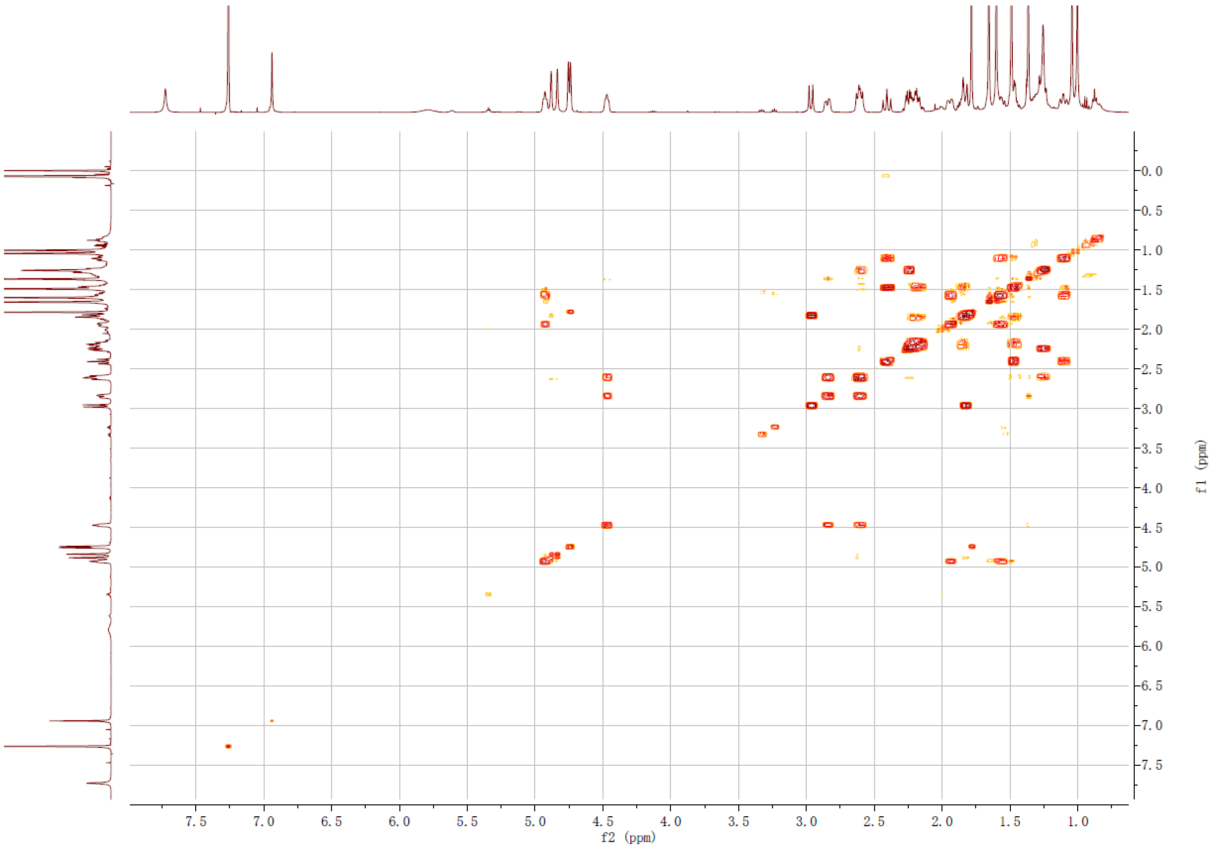

Figure S18. ^{1}H-^{1}H COSY spectrum of **2**.

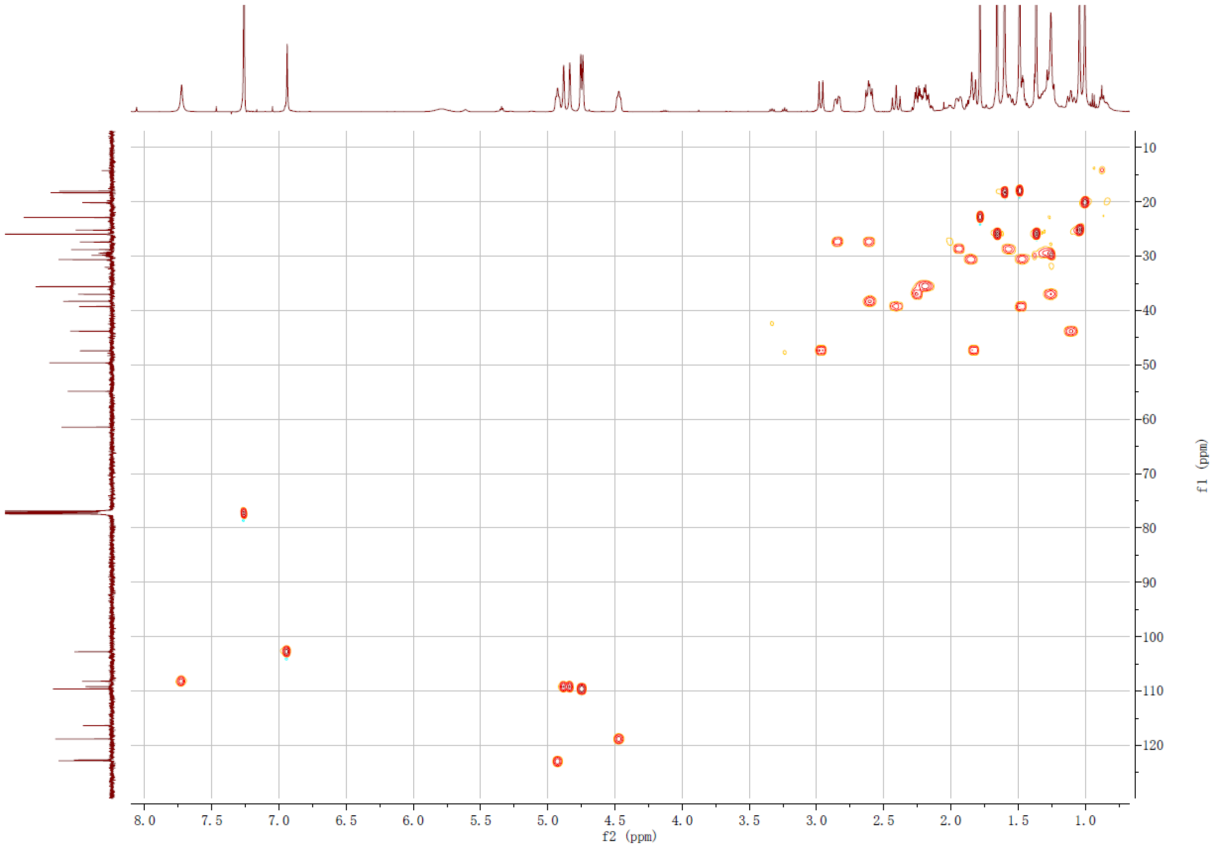

Figure S19. HSQC spectrum of **2.**

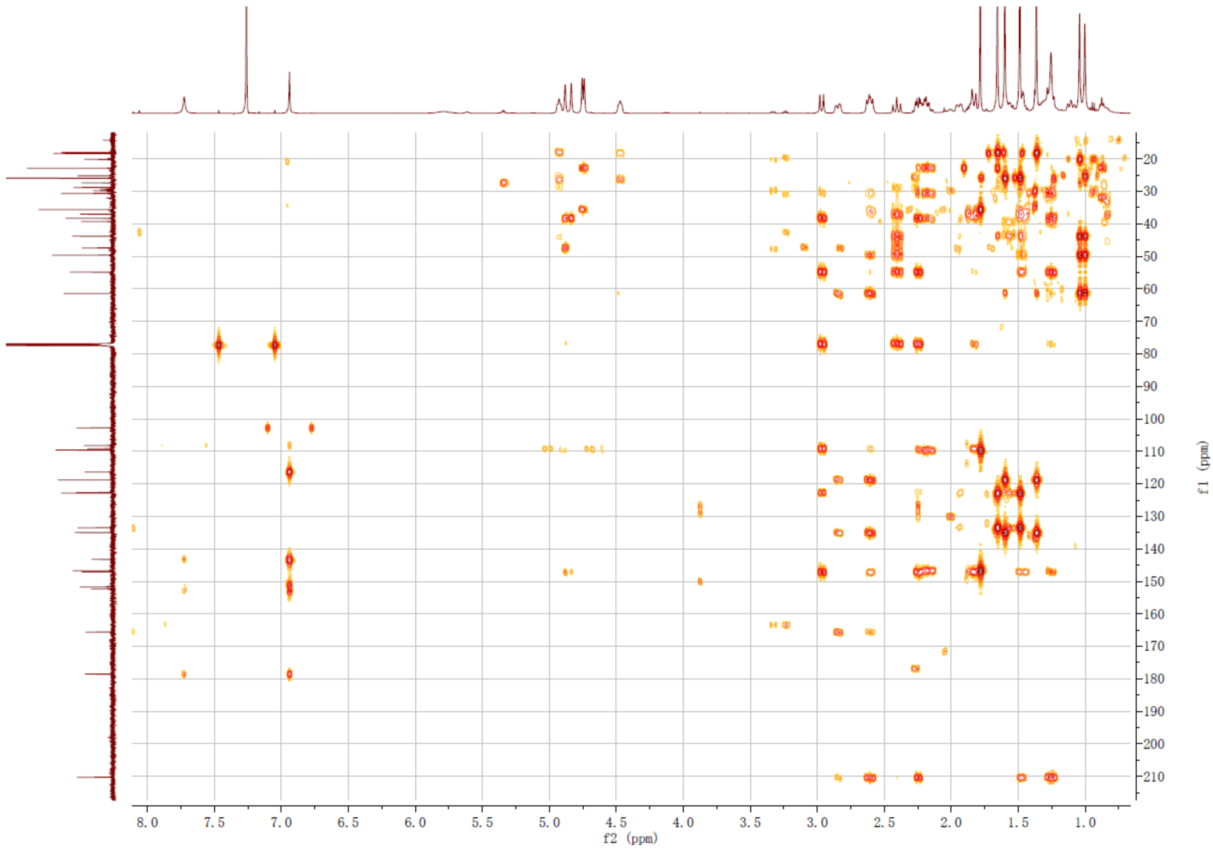

Figure S20. HMBC spectrum of **2**.

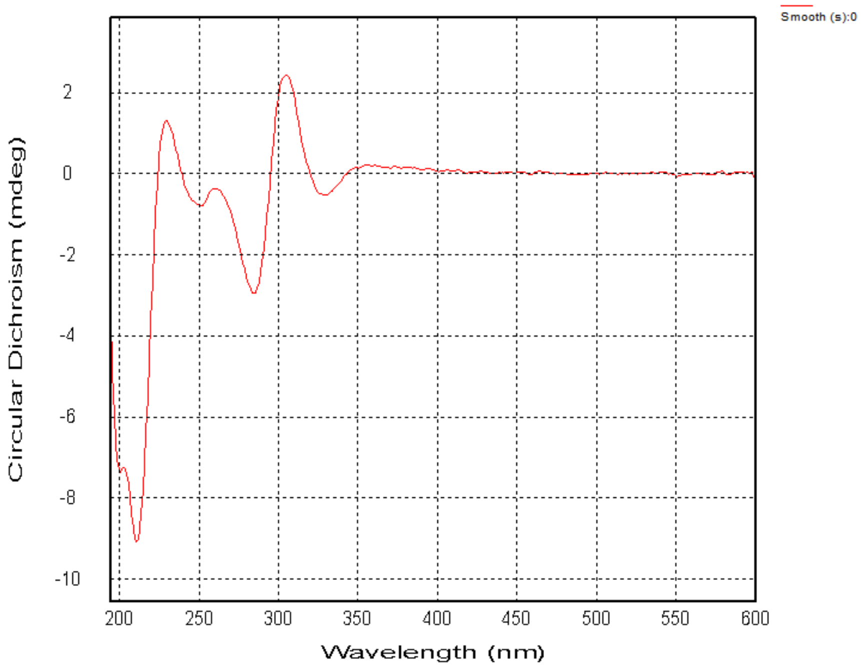

Figure S21. ECD spectrum of **3**. Concentration: 0.1231 mg/mL in MeOH; Pathlength: 1 mm.

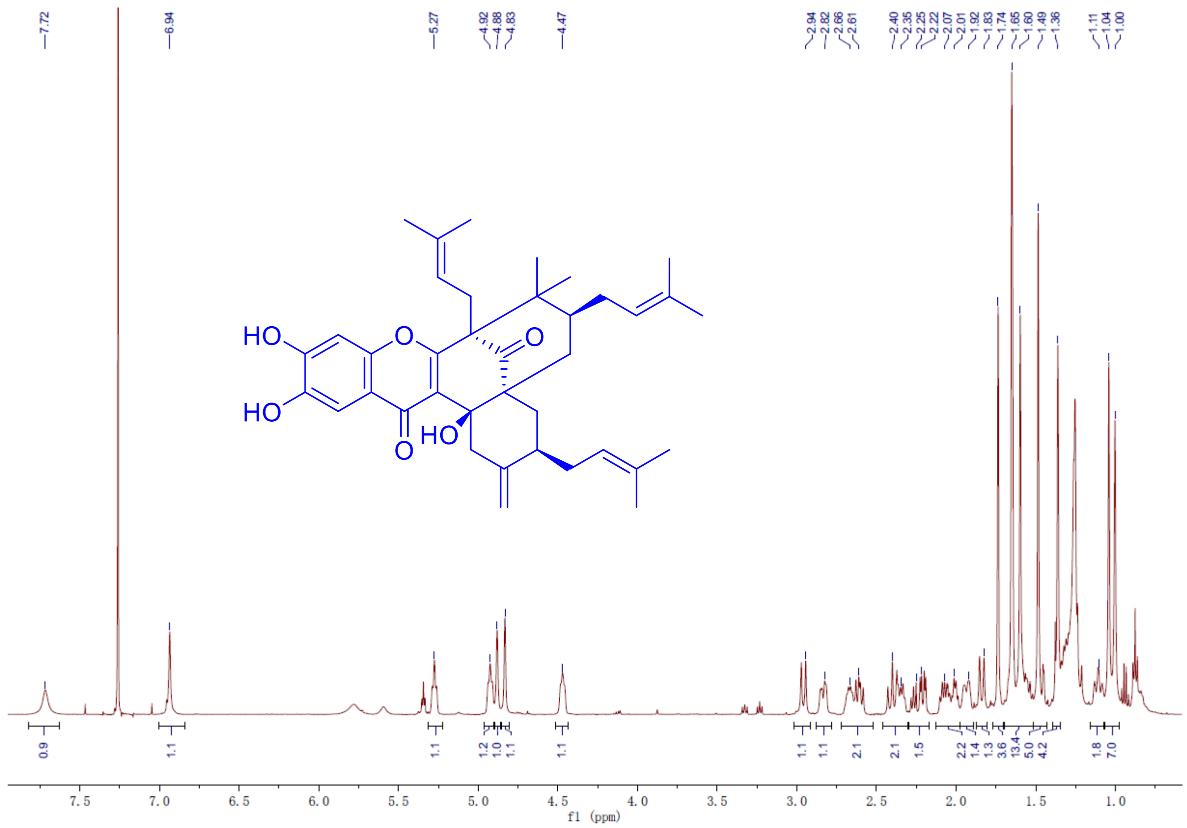

Figure S22. ^{1}H NMR (600 MHz) spectrum of **3** in $CDCl_3$.

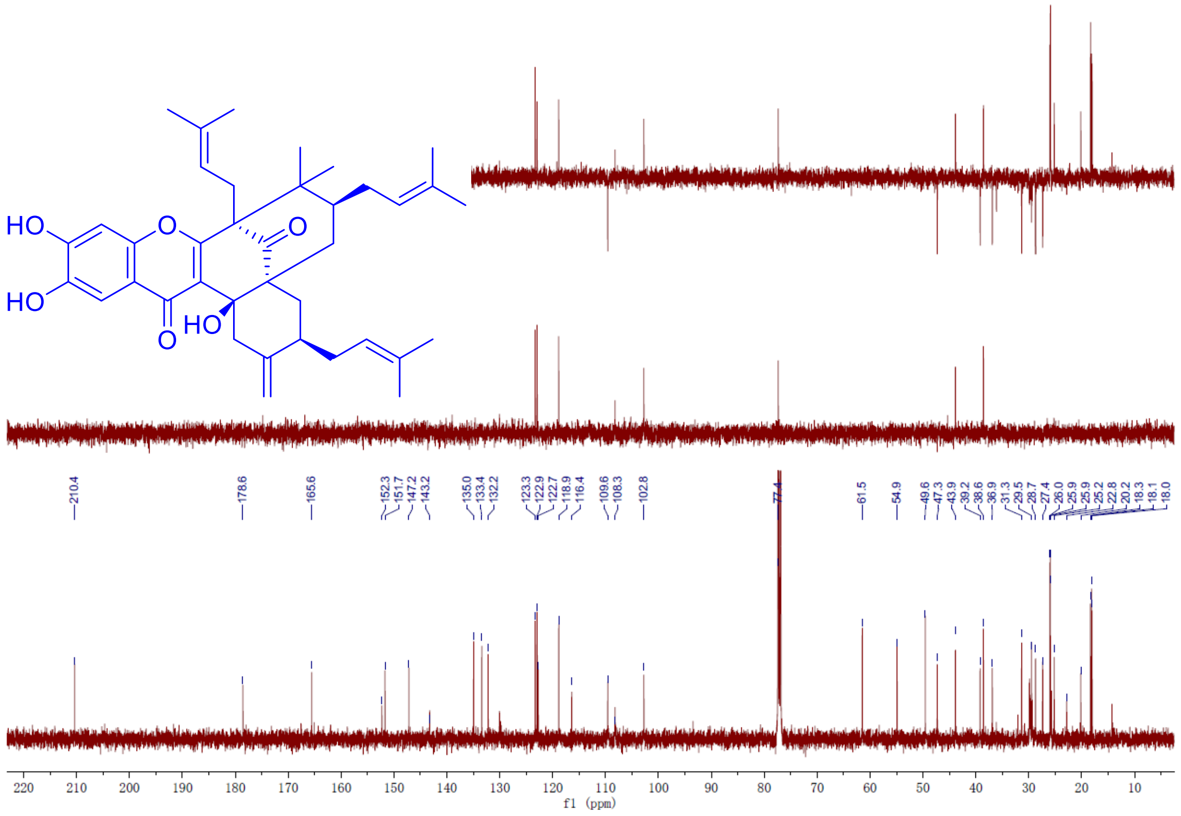

Figure S23. ^{13}C (150 MHz) and DEPT spectra of **3** in $CDCl_3$.

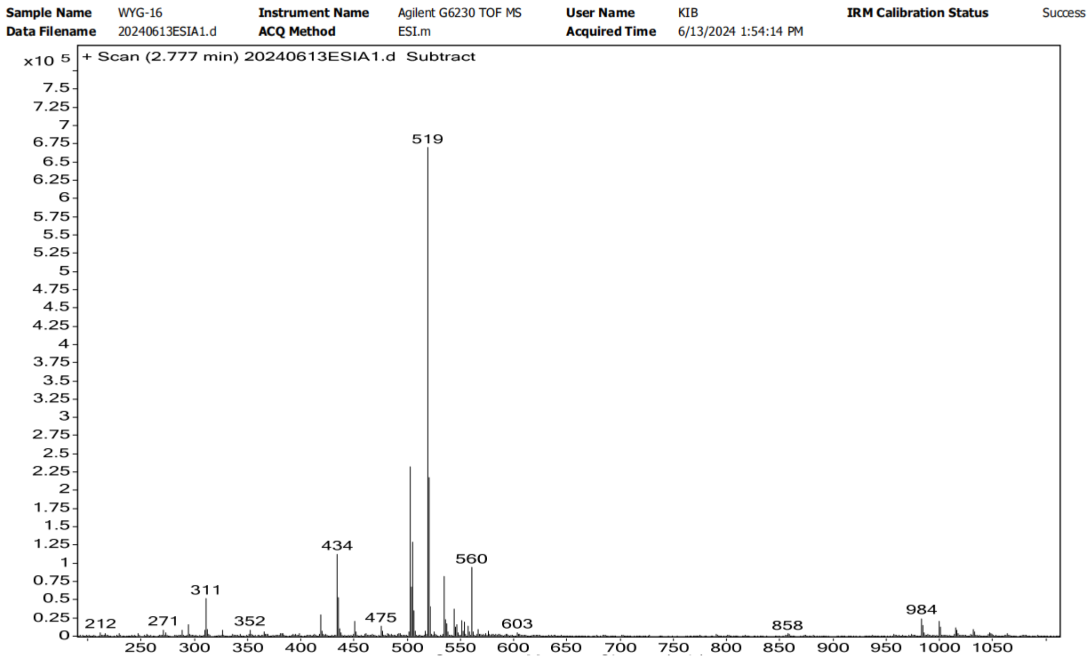

Figure S24. ESIMS of garsubelone C (**4**).

Qualitative Analysis Report

**Data Filename**	20240613ESIA1.d	**Sample Name**	WYG-16
**Sample Type**	Sample	**Position**	
**Instrument Name**	Agilent G6230 TOF MS	**User Name**	KIB
**Acq Method**	ESI.m	**Acquired Time**	6/13/2024 1:54:14 PM
**IRM Calibration Status**	Success	**DA Method**	ESI.m
**Comment**			

**Sample Group**		**Info.**
**Acquisition SW**	6200 series TOF/6500 series	
**Version**	Q-TOF B.05.01 (B5125.2)	

User Spectra

Fragmentor Voltage	Collision Energy	Ionization Mode
200	0	ESI

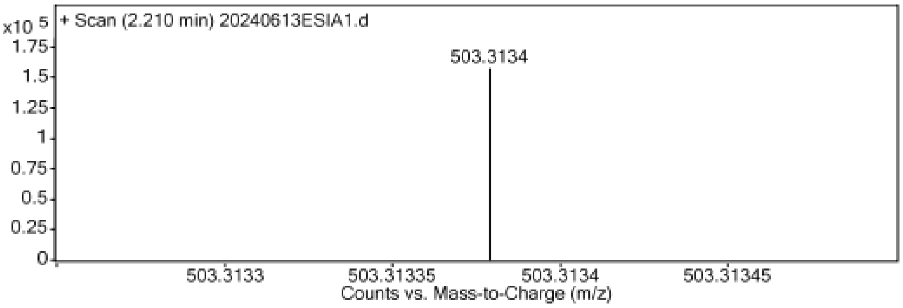

Peak List

m/z	z	Abund	Formula	Ion
212.1183	1	219538.69		
503.3134	1	157076.28	C31 H44 Na O4	M+
519.3069	1	116232.55		
983.6371	1	203346.3		
984.6401	1	130852.9		
999.6313	1	212650.31		
1000.6346	1	137080.02		
1001.6227	1	111809.39		
1015.6265	1	200716.7		
1016.6297	1	129039.59		

Formula Calculator Element Limits

Element	Min	Max
C	0	200
H	0	400
O	0	10
Na	1	1

Formula Calculator Results

Formula	CalculatedMass	Mz	Diff.(mDa)	Diff. (ppm)	DBE
C31 H44 Na O4	503.3137	503.3134	0.3	0.7	9.5

--- End Of Report ---

Figure S25. HRESIMS of garsubelone C (**4**).

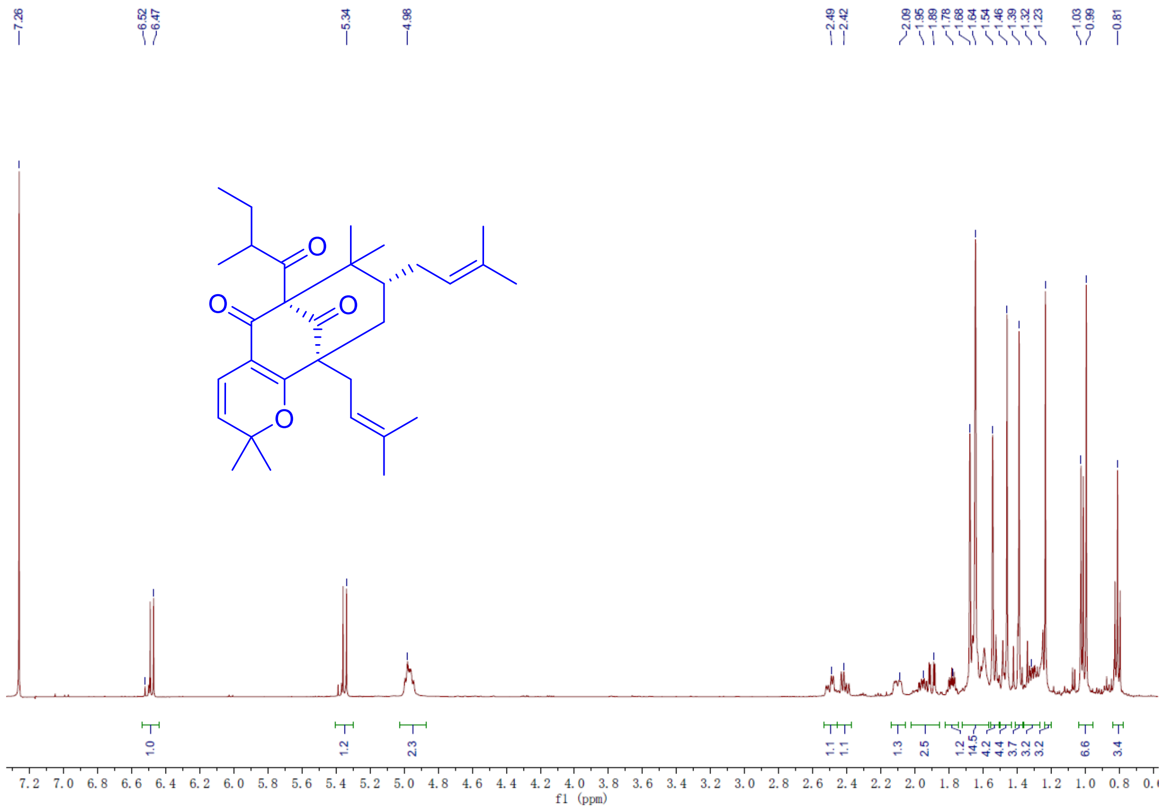

Figure S26. ^{1}H NMR (600 MHz) spectrum of **4** in $CDCl_3$.

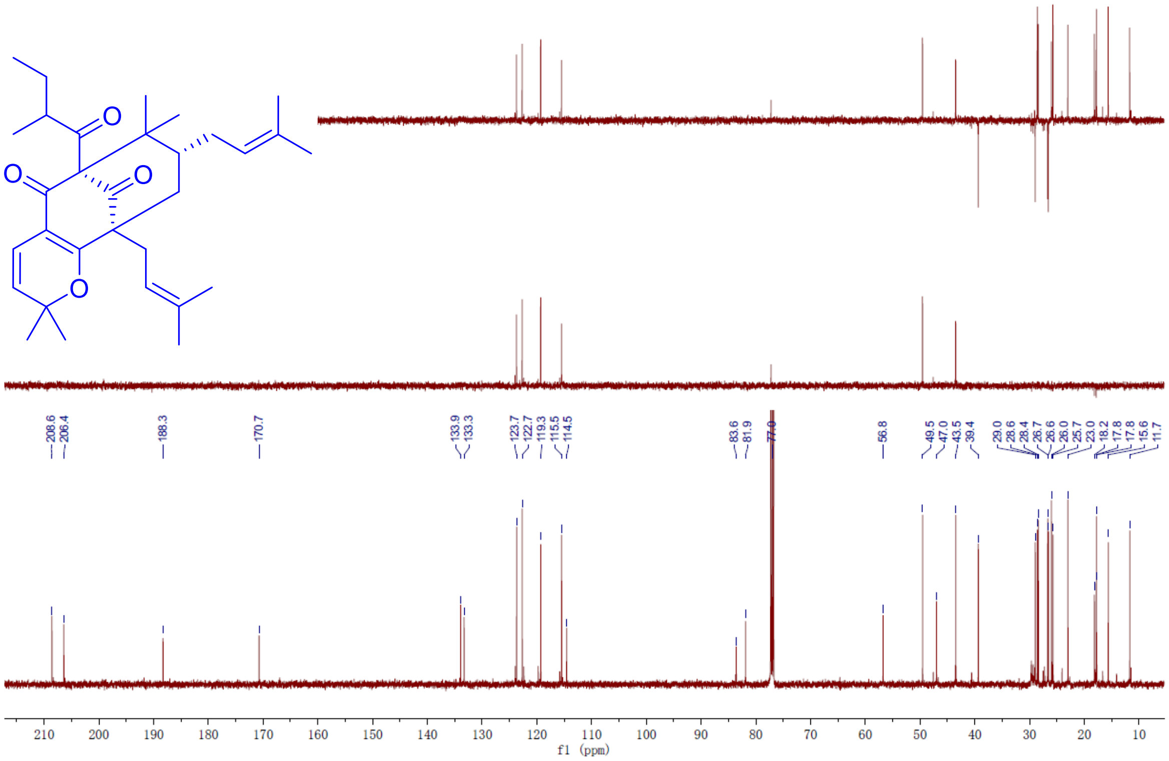

Figure S27. ^{13}C (150 MHz) and DEPT spectra of **4** in $CDCl_3$.

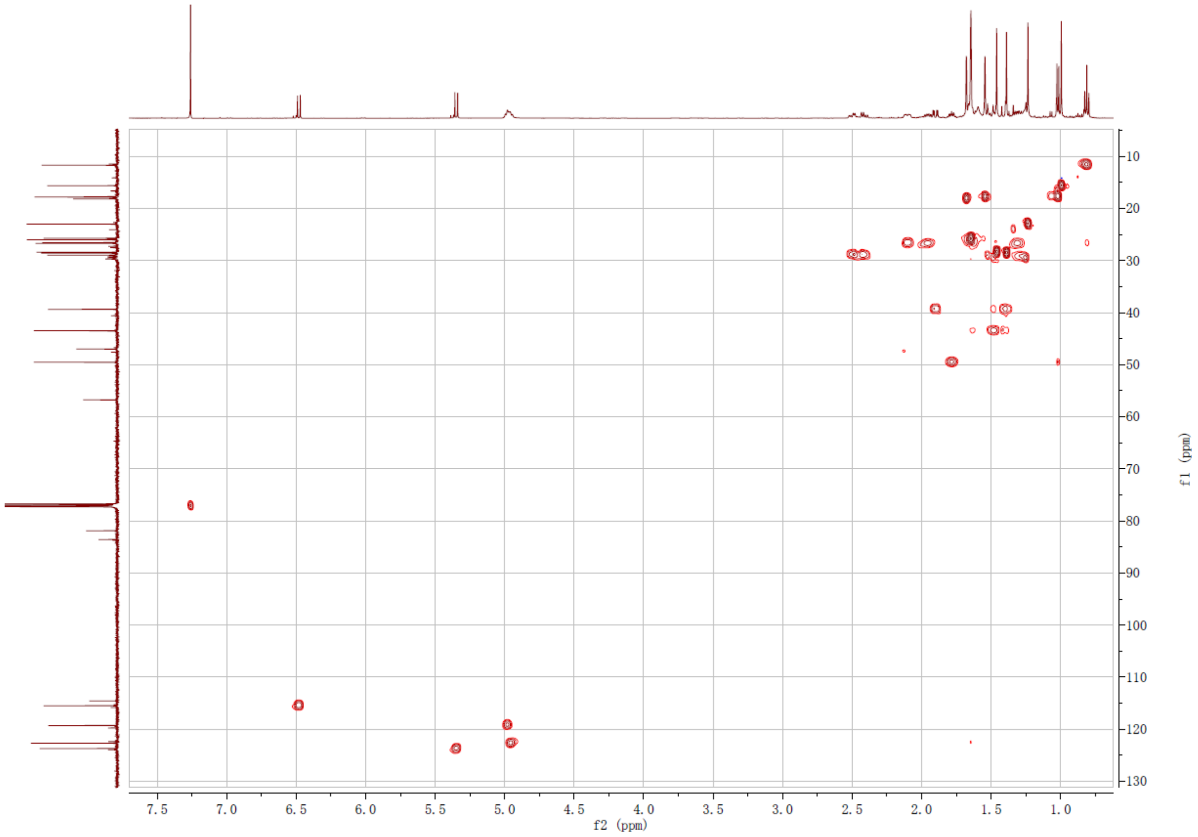

Figure S28. HSQC spectrum of **4**.

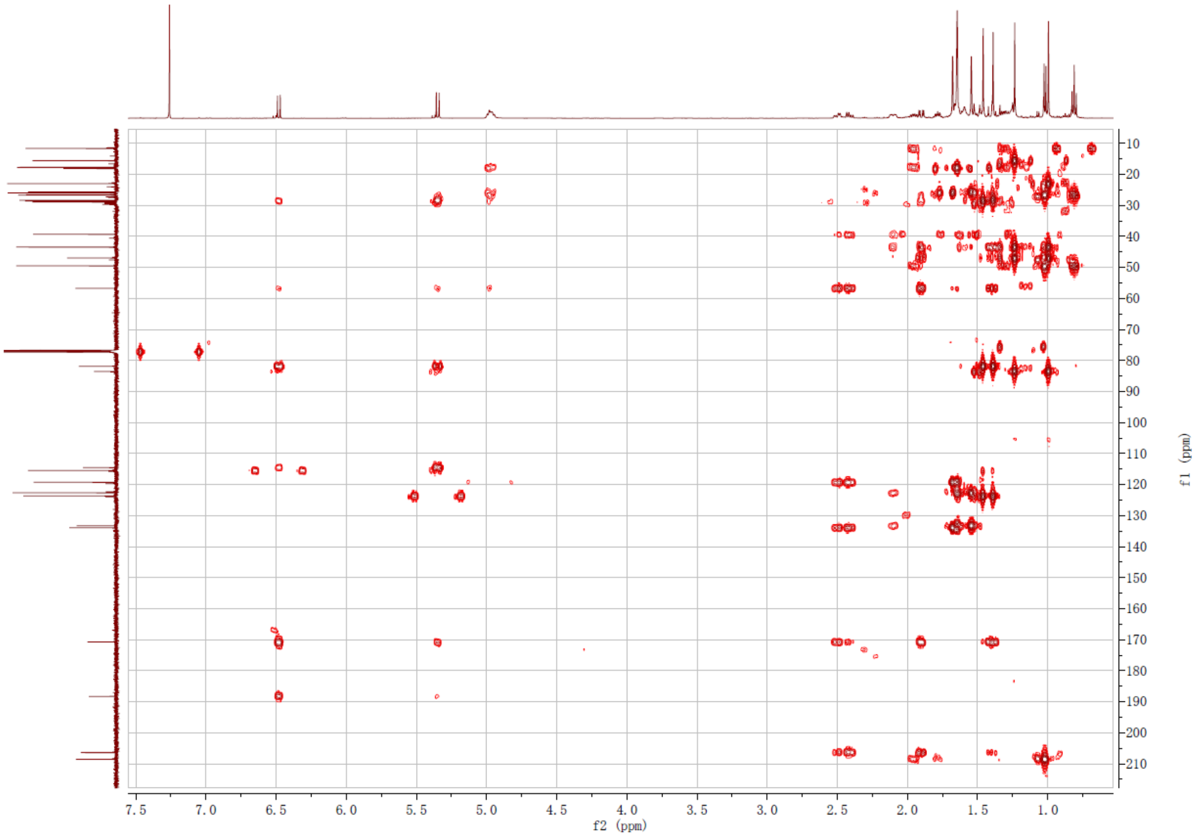

Figure S29. HMBC spectrum of **4**.

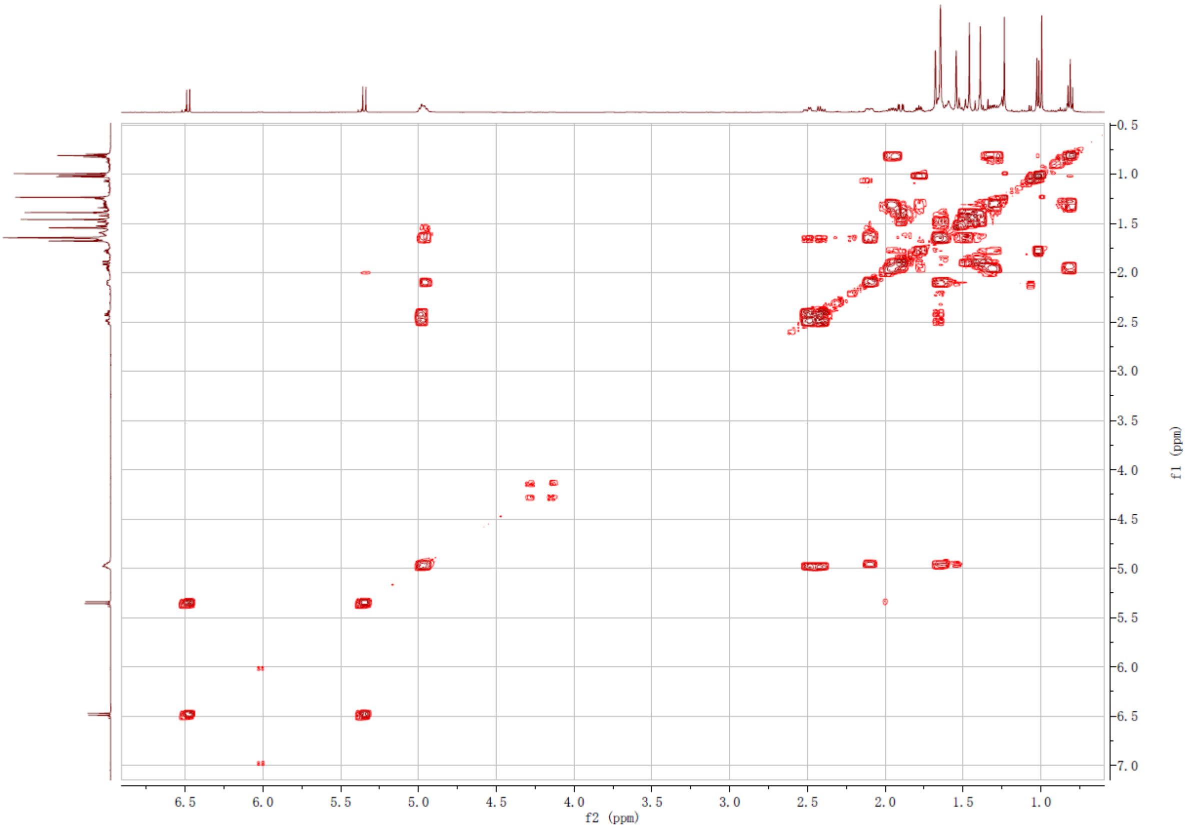

Figure S30. ^{1}H-^{1}H COSY spectrum of **4**.

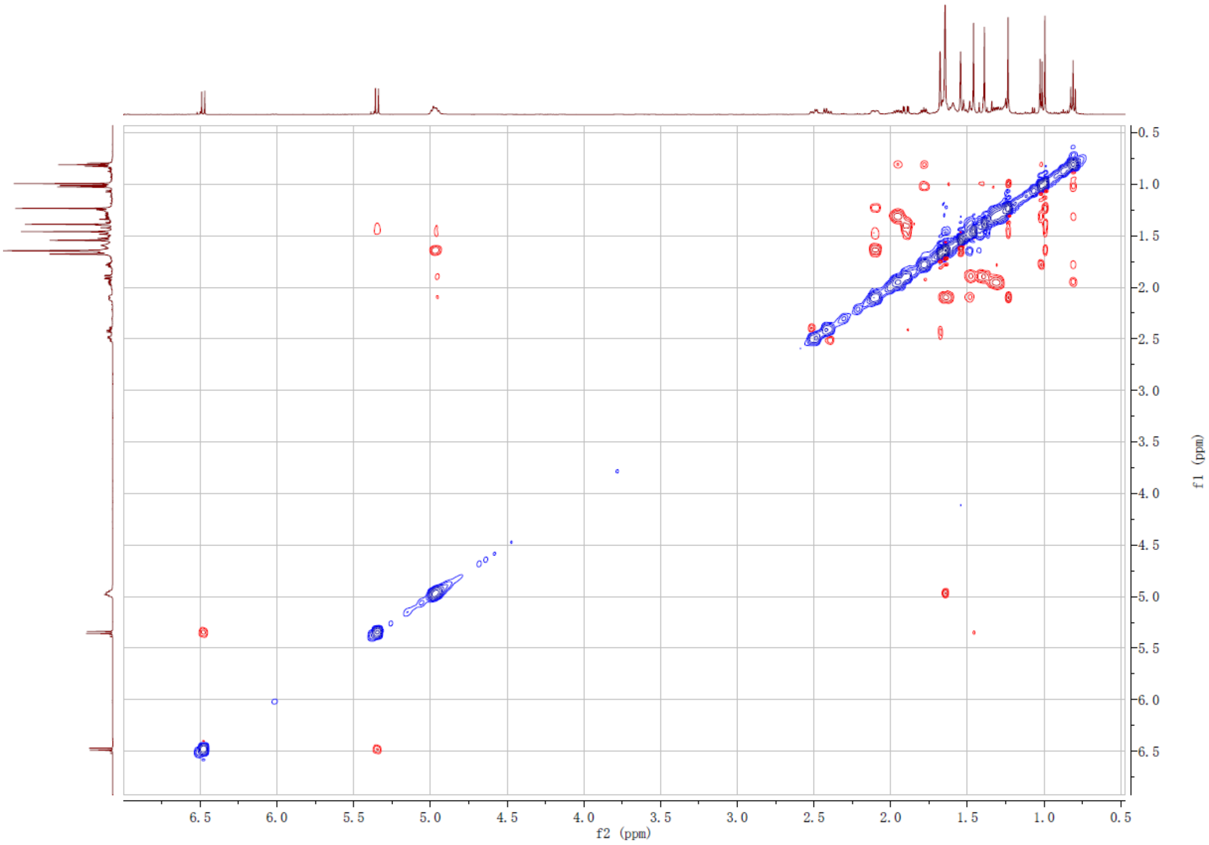

Figure S31. NOESY spectrum of **4**.

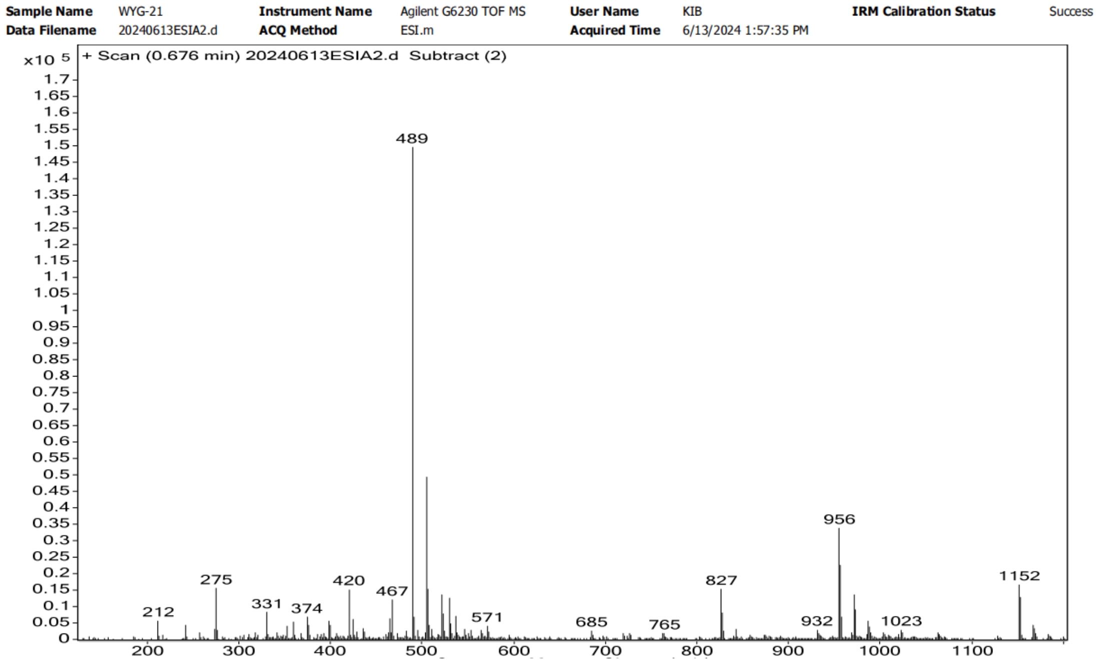

Figure S32. ESIMS of garsubelone D (**5**).

Qualitative Analysis Report

**Data Filename**	20240613ESIA2.d	**Sample Name**	WYG-21
**Sample Type**	Sample	**Position**	
**Instrument Name**	Agilent G6230 TOF MS	**User Name**	KIB
**Acq Method**	ESI.m	**Acquired Time**	6/13/2024 1:57:35 PM
**IRM Calibration Status**	Success	**DA Method**	ESI.m
**Comment**			

**Sample Group**		**Info.**
**Acquisition SW**	6200 series TOF/6500 series	
**Version**	Q-TOF B.05.01 (B5125.2)	

User Spectra

Fragmentor Voltage	Collision Energy	Ionization Mode
200	0	ESI

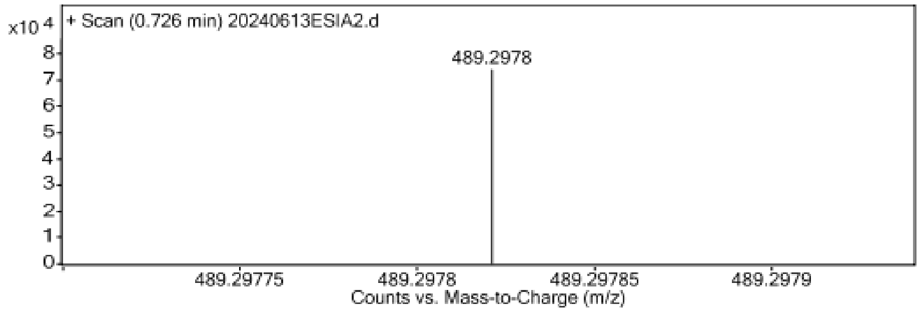

Peak List

m/z	z	Abund	Formula	Ion
121.0509	1	77897.16		
293.1742	1	13265.6		
489.2978	1	74017.27	C30 H42 Na O4	M+
490.3005	1	21139.67	C30 H42 Na O4	M+
505.2901	1	19429.69		
530.3165	1	10925.79		
922.0098	1	142835.63		
923.0123	1	20920.94		
955.6051	1	16221.68		
956.6087	1	10234.62		

Formula Calculator Element Limits

Element	Min	Max
C	0	200
H	0	400
O	0	10
Na	1	1

Formula Calculator Results

Formula	CalculatedMass	Mz	Diff.(mDa)	Diff. (ppm)	DBE
C30 H42 Na O4	489.2981	489.2978	0.3	0.6	9.5

--- End Of Report ---

Figure S33. HRESIMS of garsubelone D (**5**).

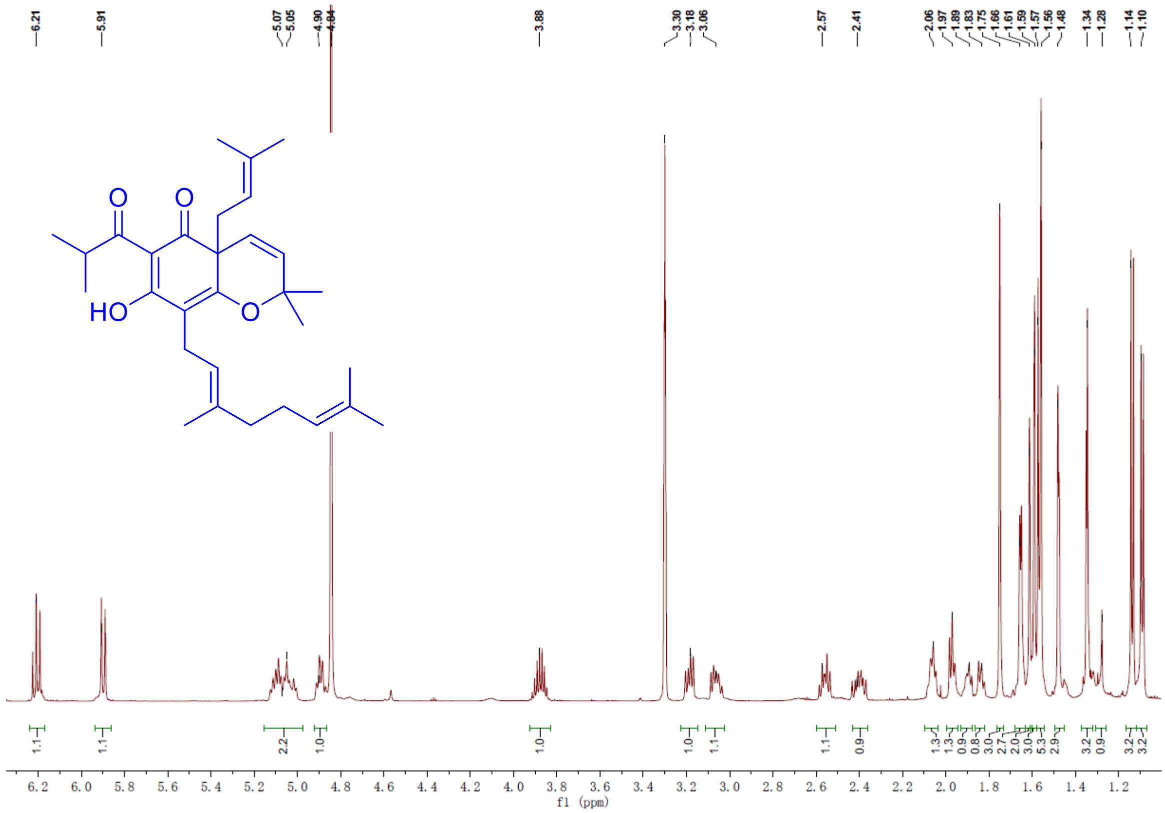

Figure S34. ^{1}H NMR (600 MHz) spectrum of **5** in methanol-d_4.

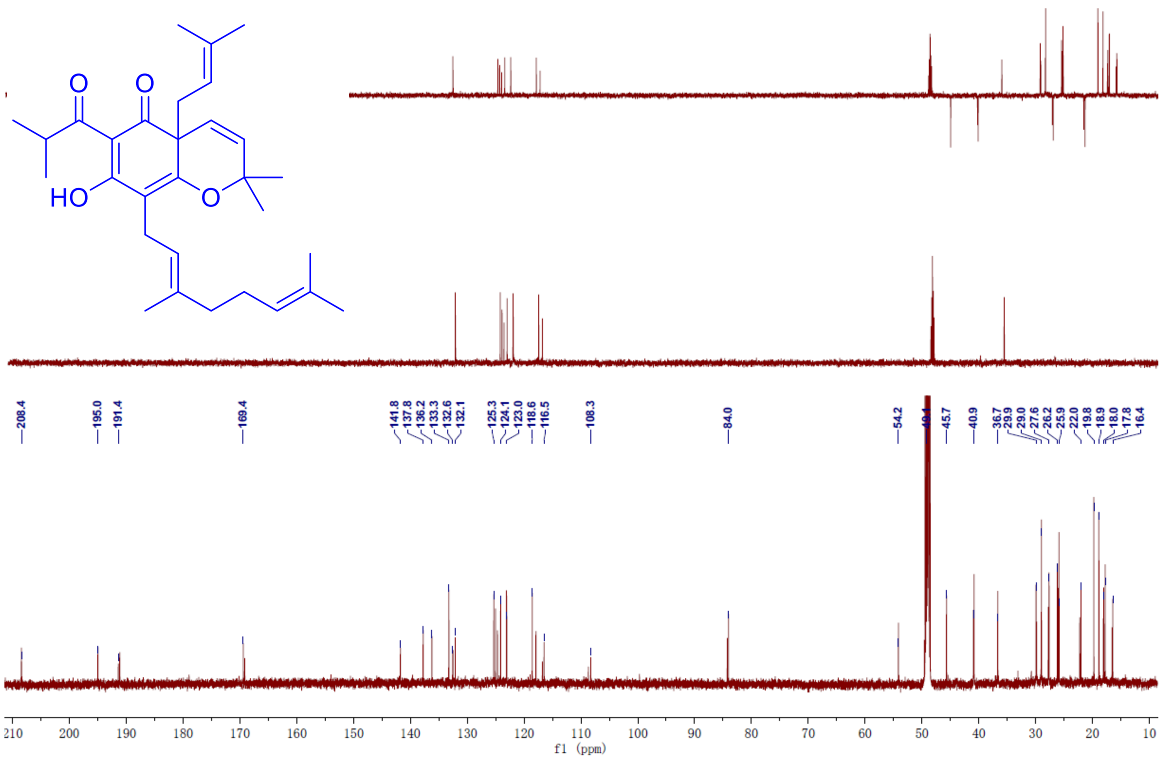

Figure S35. ^{13}C (150 MHz) and DEPT spectra of **5** in methanol-d_4.

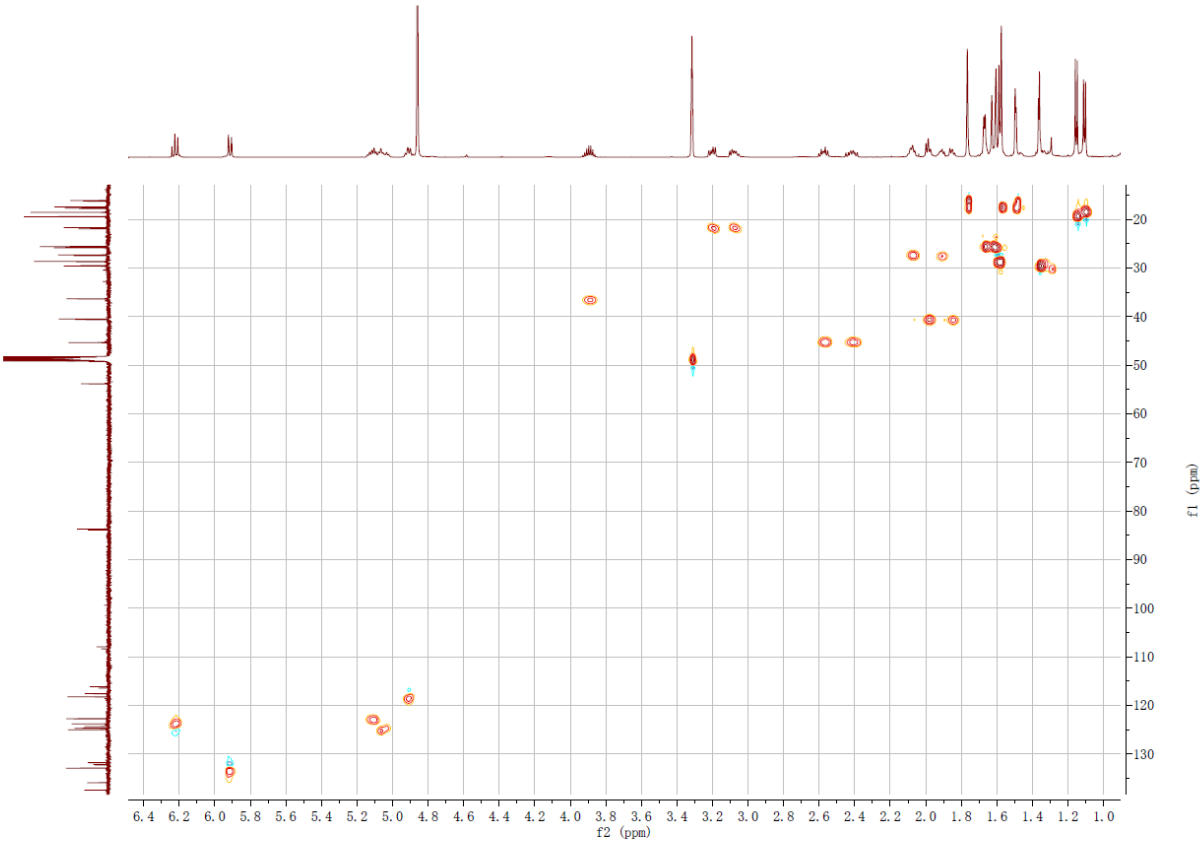

Figure S36. HSQC spectrum of **5**.

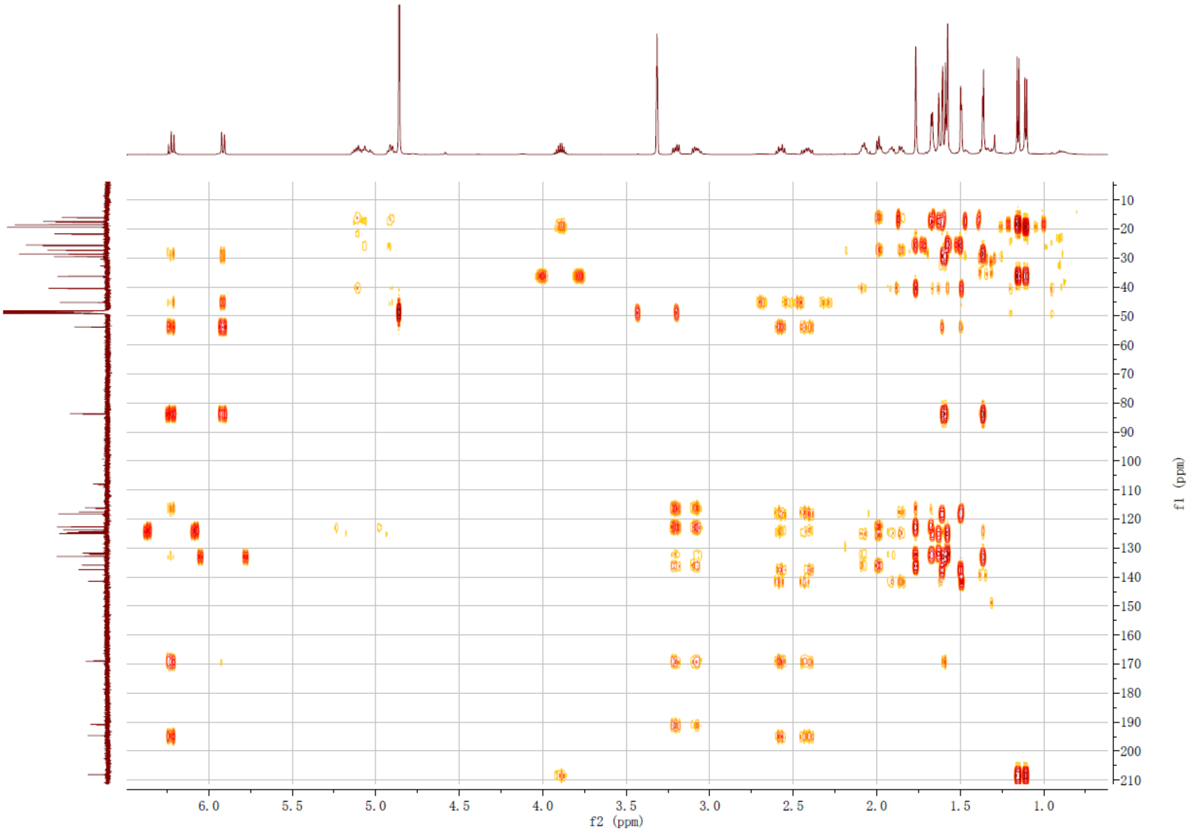

Figure S37. HMBC spectrum of **5**.

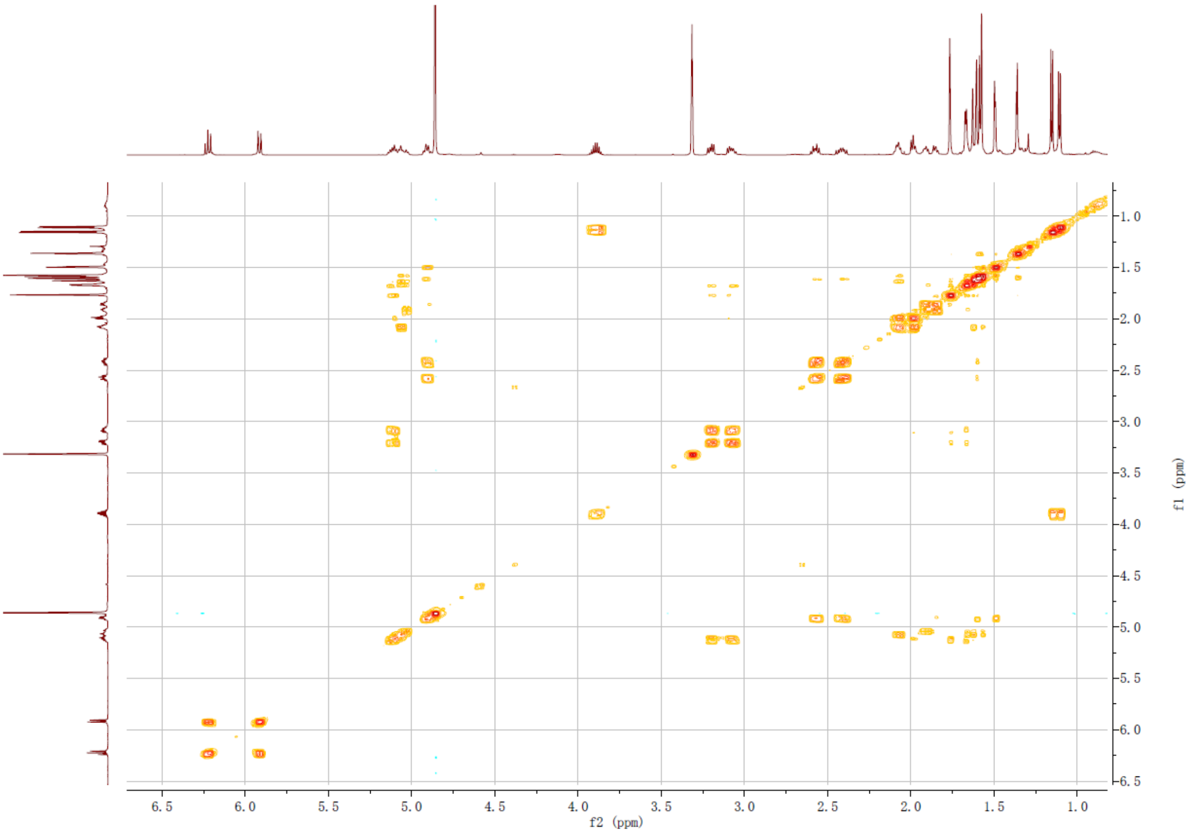

Figure S38. ^{1}H-^{1}H COSY spectrum of **5**.

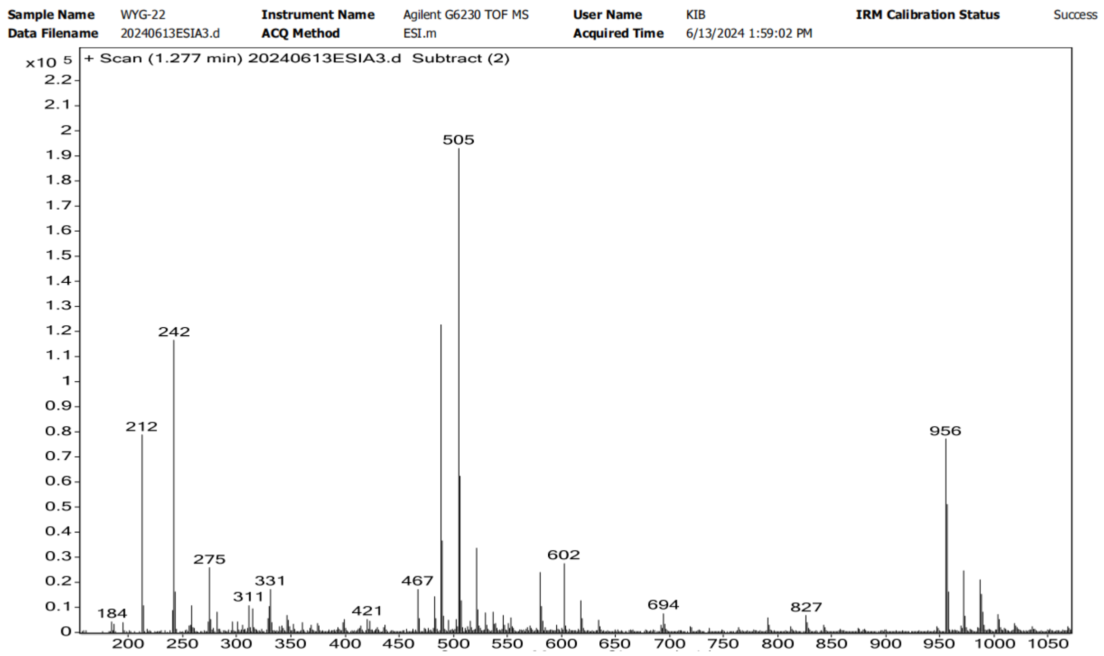

Figure S39. ESIMS of garsubelone E (**6**).

Qualitative Analysis Report

**Data Filename**	20240613ESIA3.d	**Sample Name**	WYG-22
**Sample Type**	Sample	**Position**	
**Instrument Name**	Agilent G6230 TOF MS	**User Name**	KIB
**Acq Method**	ESI.m	**Acquired Time**	6/13/2024 1:59:02 PM
**IRM Calibration Status**	Success	**DA Method**	ESI.m
**Comment**			

**Sample Group**		**Info.**
**Acquisition SW Version**	6200 series TOF/6500 series Q-TOF B.05.01 (B5125.2)	

User Spectra

Fragmentor Voltage	Collision Energy	Ionization Mode
200	0	ESI

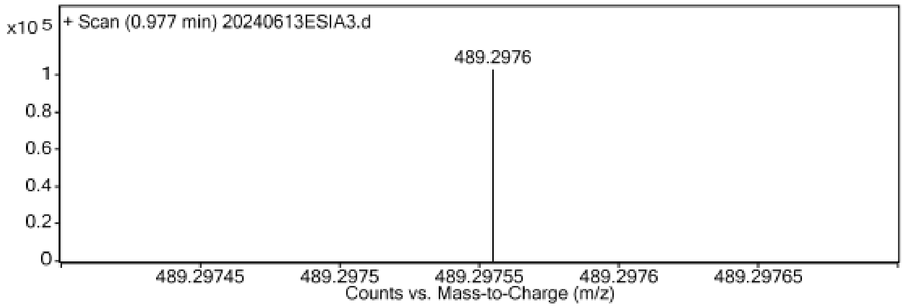

Peak List

m/z	z	Abund	Formula	Ion
121.0509		42401.21		
212.118	1	64992.43		
242.2842	1	142286.61		
489.2976	1	102549.18	C30 H42 Na O4	M+
505.2923	1	185893.42		
506.2957	1	61055.15		
521.285	1	33358.21		
922.0098	1	83581.78		
955.6052	1	60864.03		
956.6087	1	39655.05		

Formula Calculator Element Limits

Element	Min	Max
C	0	200
H	0	400
O	0	10
Na	1	1

Formula Calculator Results

Formula	CalculatedMass	Mz	Diff.(mDa)	Diff. (ppm)	DBE
C30 H42 Na O4	489.2981	489.2976	0.5	1.0	9.5

--- End Of Report ---

Figure S40. HRESIMS of garsubelone E (**6**).

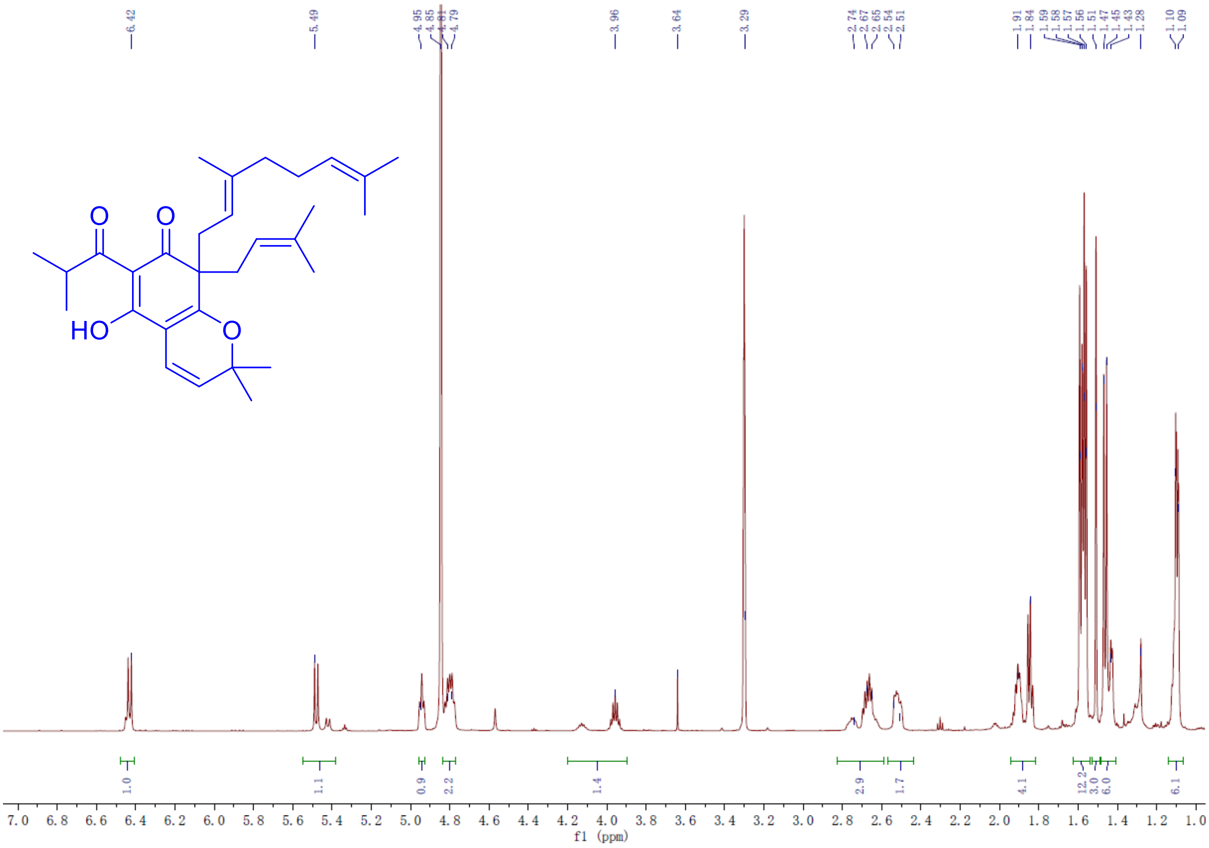

Figure S41. ^{1}H NMR (600 MHz) spectrum of **6** in methanol-d_4.

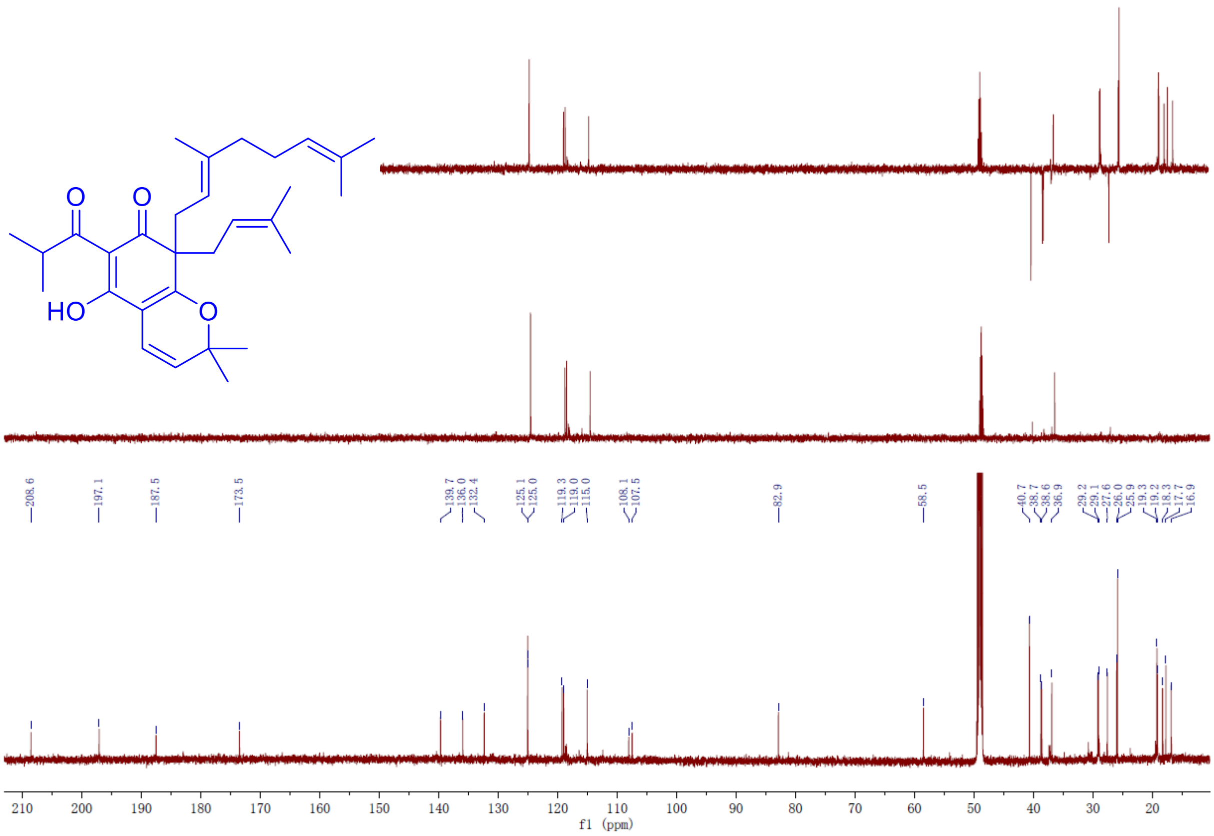

Figure S42. ^{13}C (150 MHz) and DEPT spectra of **6** in methanol-d_4.

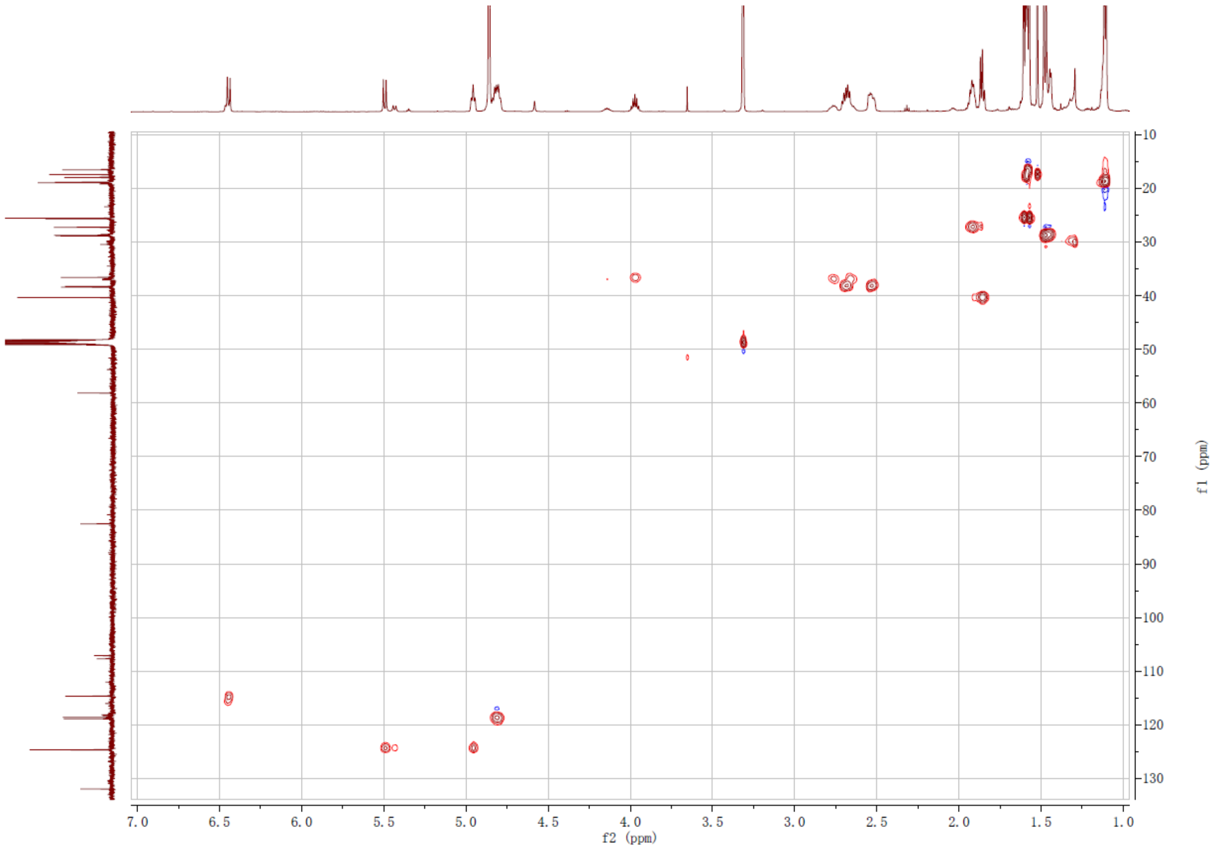

Figure S43. HSQC spectrum of **6**.

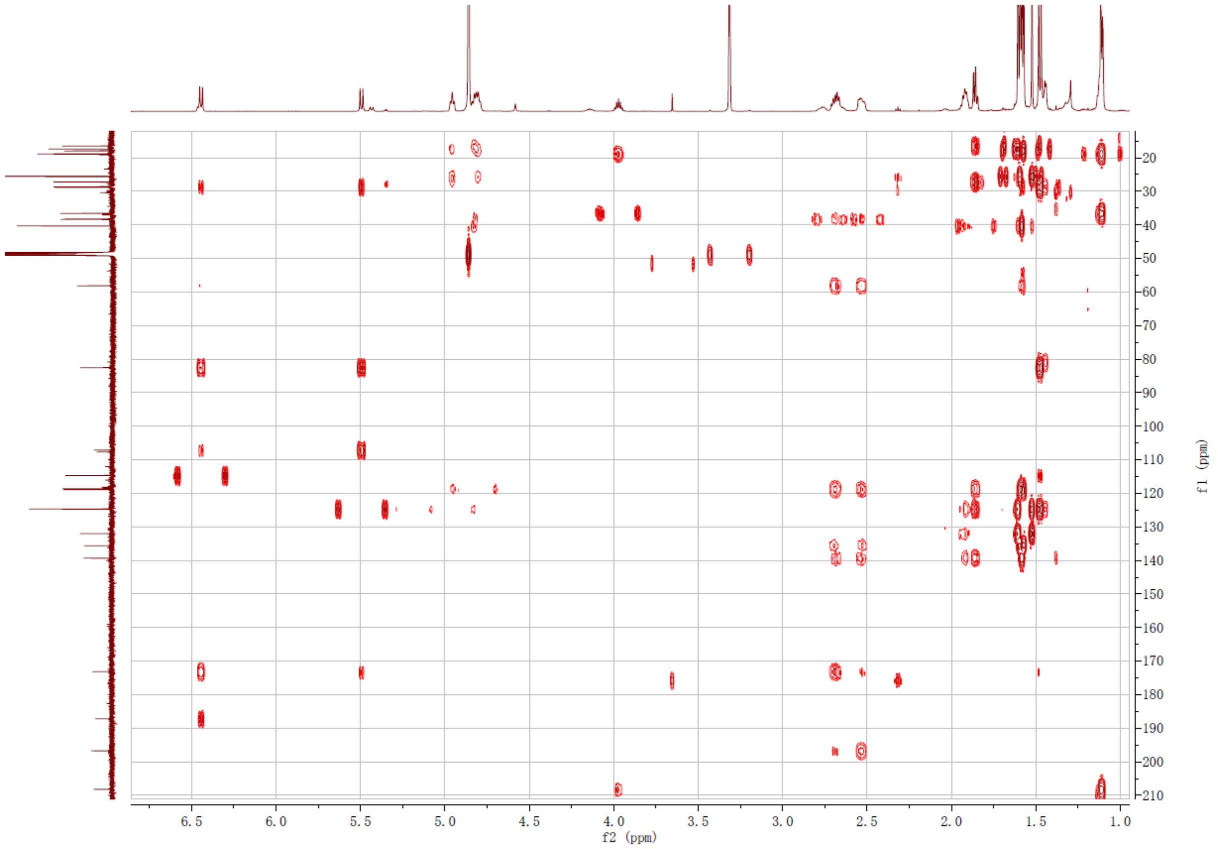

Figure S44. HMBC spectrum of **6**.

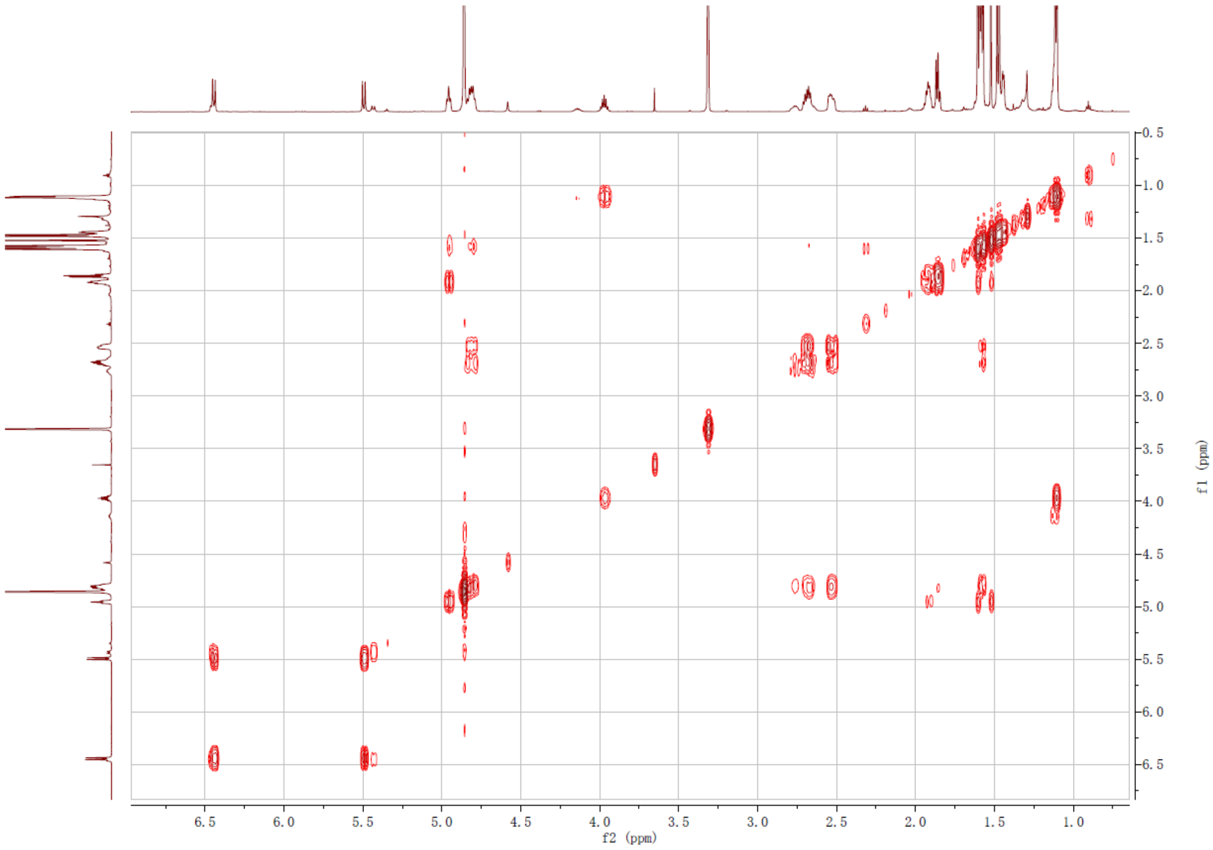

Figure S45. ^{1}H-^{1}H COSY spectrum of **6**.
